# Food–Medicine Homologous Qiongyu Gao Attenuates Skin Photoaging by Remodeling Gut Microbiota and Restoring Mitochondrial Energy Metabolism

**DOI:** 10.3390/foods15162824

**Published:** 2026-08-13

**Authors:** Ziyi Yang, Bingchen Han, Ying Chen, Youqing Wang, Yuzhen Huang, Jiali Ran, Jianjun Liang, Xiaobo Zeng, Haiying Wang

**Affiliations:** 1 College of Life Sciences, South-Central Minzu University, Wuhan 430074, China; 2024010062@mail.scuec.edu.cn (Z.Y.); 2023010071@mail.scuec.edu.cn (B.H.); 2023120715@mail.scuec.edu.cn (Y.C.); 2024120510@mail.scuec.edu.cn (Y.W.); 2025120767@mail.scuec.edu.cn (Y.H.); 2021120425@mail.scuec.edu.cn (J.R.); 8800154@mail.scuec.edu.cn (J.L.); 2 School of Pharmaceutical Science, Wuhan University, 185 Donghu Road, Wuhan 430071, China

**Keywords:** functional food, food–medicine homologous formula, Qiongyu Gao, gut microbiota, gut–skin axis, serum metabolomics, mitochondrial energy metabolism, skin photoaging

## Abstract

Bioactive food ingredients that regulate the gut microbiota are promising dietary strategies for supporting systemic health, but their roles in skin photoaging remain insufficiently defined. Qiongyu Gao (QYG), a classical food–medicine homologous formula composed of Rehmanniae Radix, *Panax ginseng*, and *Poria cocos*, was evaluated as an oral functional food candidate for UV-induced skin photoaging. QYG was chemically characterized by HPLC and UPLC–QTOF–MS/MS. Young and aged mice were subjected to D-galactose plus UVA/UVB exposure and orally administered QYG, followed by skin transcriptomics, gut microbiota sequencing, serum metabolomics, and validation in UVB-injured primary dermal fibroblasts. QYG alleviated wrinkle formation, epidermal thickening, oxidative stress, inflammation, extracellular matrix degradation, collagen disorganization, and hyaluronic acid loss. Multi-omics analysis showed that QYG selectively remodeled gut microbiota, enriching *Lactobacillus*-, *Bifidobacterium*-, and *Akkermansia*-associated taxa, regulated serum metabolites related to energy and lipid metabolism, and enriched mitochondrial energy metabolism-related pathways in photoaged skin. In fibroblasts, QYG-containing serum restored mitochondrial membrane potential, reduced ROS accumulation and cellular senescence, and regulated AMPK/PGC-1α-associated markers. These findings support QYG as a promising food–medicine homologous functional food candidate for skin health maintenance through gut microbiota-associated systemic metabolic regulation.

## 1. Introduction

Functional foods and bioactive food ingredients have attracted increasing attention because of their potential to support human health beyond basic nutrition. In recent years, the gut microbiota has been recognized as an important mediator linking dietary intake with host metabolism and immune homeostasis [1,2]. Various dietary bioactive components, particularly plant-derived dietary fibers and polysaccharides, can interact with intestinal microorganisms and influence microbial composition, microbial metabolites, and downstream host responses [3]. Therefore, functional foods capable of modulating the gut microbiota are regarded as promising dietary strategies for improving systemic health and preventing age-related functional decline.

Skin health is closely connected with systemic metabolic and immune status. Skin photoaging, a chronic degenerative process mainly induced by prolonged ultraviolet (UV) exposure, has become an important public health and cosmetic concern. More than 80% of visible facial aging signs, including wrinkles and loss of elasticity, are associated with UV exposure, and the prevalence of photoaging can exceed 90% among elderly populations in regions with high UV radiation [4,5,6]. Mechanistically, UV radiation induces excessive reactive oxygen species (ROS) generation, matrix metalloproteinase (MMP) activation, and DNA photolesion formation, thereby promoting epidermal thickening, dermal collagen degradation, elastic fiber disorganization, and impaired skin repair [7,8]. Thus, safe and sustainable oral functional food interventions that enhance skin stress resistance and tissue repair are of increasing interest for skin health maintenance.

In addition to oxidative stress, inflammation, and DNA damage, mitochondrial dysfunction has increasingly been recognized as an important mechanism involved in skin aging and photoaging. Continuous UV exposure can impair mitochondrial membrane potential, disrupt respiratory activity, amplify ROS production, and promote apoptosis and cellular senescence [9,10]. As mitochondria are central hubs of ATP generation, redox regulation, inflammatory responses, and stress adaptation, impaired mitochondrial energy metabolism may aggravate oxidative injury and weaken the repair capacity of photoaged skin [11]. Mitochondrial energy metabolism depends largely on oxidative phosphorylation, the tricarboxylic acid (TCA) cycle, and stress-adaptive metabolic signaling. TCA cycle intermediates not only serve as substrates for ATP production but also participate in the regulation of cellular function and stress responses [12]. Therefore, maintaining mitochondrial energy homeostasis may be important for improving the resistance and repair capacity of UV-damaged skin.

For orally administered functional food interventions, the gut–skin axis provides a plausible route linking dietary intake, gut microbiota regulation, systemic metabolism, and skin physiology. A balanced gut microbiota contributes to host metabolic and immune homeostasis, whereas gut microbiota dysbiosis may aggravate oxidative stress and inflammatory damage in peripheral tissues, including the skin [1,13,14,15,16]. Recent evidence also suggests bidirectional communication between skin injury and intestinal microbial homeostasis [17]. In particular, dietary carbohydrates and plant-derived polysaccharides can interact with gut microbes and influence microbial metabolic functions [3]. Thus, gut microbiota remodeling may represent an important upstream mechanism by which oral functional foods regulate systemic metabolism and contribute to skin protection.

Qiongyu Gao (QYG), also known as Kyung-ok-ko in Korea, is a classical food–medicine homologous formula generally traced to *Hong’s Collection of Effective Prescriptions* (*Hong Shi Ji Yan Fang*) and also documented in *Compendium of Materia Medica* (*Ben Cao Gang Mu*). It is composed of Rehmanniae Radix, *Panax ginseng*, and *Poria cocos*. These three ingredients are included in official Chinese food-related regulatory lists as substances traditionally used as both food and medicine and/or substances permitted for use in health foods (Ministry of Health of the People’s Republic of China, 2002). Traditionally, QYG has long been used as a tonic preparation for nourishing vitality and delaying age-related decline. Modern studies suggest that QYG and QYG-related preparations possess multi-component and multi-target characteristics, with reported antioxidant, anti-inflammatory, neuroprotective, immunomodulatory, and anti-fatigue effects [18,19]. However, the food-related functional mechanisms by which QYG supports skin health, particularly its potential role in UV-induced photoaging through gut microbiota remodeling, serum metabolic regulation, and mitochondrial energy metabolism, remain insufficiently characterized.

In this study, young and aged Kunming mice were subjected to combined D-galactose and UVA/UVB exposure and orally administered QYG to evaluate its protective effects against skin photoaging. HPLC and UPLC–QTOF–MS/MS were used to characterize representative chemical constituents of QYG. Skin transcriptomics, gut microbiota sequencing, serum metabolomics, and UVB-injured primary mouse dermal fibroblasts were further used to explore QYG-induced changes in gut microbiota composition, microbial metabolic potential, systemic metabolism, mitochondrial function, ROS accumulation, cellular senescence, and AMPK/PGC-1α-associated energy metabolism. This study provides preclinical evidence supporting QYG as a food–medicine homologous functional food candidate for skin health maintenance through gut microbiota-associated systemic metabolic regulation.

## 2. Materials and Methods

### 2.1. Preparation and Chemical Characterization of QYG

#### 2.1.1. Preparation of QYG Extract

The herbal materials used for QYG were commercially authenticated Chinese medicinal decoction pieces purchased from Hubei Yaosheng Traditional Chinese Medicine Co., Ltd. (Xiangyang, Hubei, China; production license No. E20200344). The formula consisted of *Rehmannia glutinosa* (Gaertn.) DC. radix, *Panax ginseng* C.A. Mey. radix et rhizoma, and *Poria cocos* (Schw.) Wolf sclerotium. According to the certificates of analysis provided by the supplier, the batch numbers were 20230201 for *Rehmannia glutinosa*, 20230201 for *Panax ginseng*, and 20230301 for *Poria cocos*. The producing areas were Henan, Jilin, and Hubei Province, China, respectively. *Rehmannia glutinosa* and *Poria cocos* complied with the standards of the Chinese Pharmacopoeia, 2020 edition, Volume I, according to the supplier certificates. The herbal materials were retained in the laboratory for traceability.

QYG was prepared according to the commercially established core herbal composition. Briefly, *Rehmannia glutinosa*, *Poria cocos*, and *Panax ginseng* were mixed at a ratio of 7:2:1. The mixture was extracted twice with eight volumes of water by reflux extraction for 4 h each time. The combined extracts were filtered, concentrated under reduced pressure, and freeze-dried to obtain the final QYG extract. The extract was stored at 4 °C until use.

#### 2.1.2. HPLC Identification and Quantification of Marker Compounds

HPLC was used to identify and quantify five marker compounds in the QYG extract, namely catalpol, rehmannioside, ginsenoside Rg1, ginsenoside Rb1, and pachymic acid. HPLC analysis was performed using an Agilent 1200 Series system equipped with a UV detector and an autosampler (Agilent Technologies, Santa Clara, CA, USA). The separation was performed on a Waters SymmetryShield RP18 column (4.6 mm × 250 mm, 5 µm; Waters Corporation, Milford, MA, USA) maintained at 30 °C. The mobile phases consisted of 0.1% phosphoric acid in water (A) and acetonitrile (B). Chromatographic separation was conducted at a flow rate of 1.0 mL/min with UV detection at 205 nm using the following gradient program: 0–7 min, 2% B; 7–20 min, 2–45% B; 20–30 min, 45–70% B; 30–35 min, 70–80% B; 35–45 min, 80–90% B; 45–45.1 min, 90–2% B; and 45.1–55 min, 2% B. Reference standards for these five compounds were purchased from Chengdu Zhibiao Pure Biotechnology Co., Ltd. (Chengdu, China). The corresponding chromatographic peaks in the QYG extract were identified by matching their retention times with those of the reference standards analyzed under identical chromatographic conditions. Quantification was performed using the external-standard method based on calibration curves constructed from serial concentrations of each reference compound.

#### 2.1.3. UPLC–QTOF–MS/MS Analysis

For the analysis of the chemical composition of QYG, a Vanquish UHPLC system (Thermo Fisher Scientific, Waltham, MA, USA) coupled to an Orbitrap Exploris 120 high-resolution mass spectrometer (Thermo Fisher Scientific, Waltham, MA, USA) was employed. QYG powder (10.0 mg) was accurately weighed and transferred to a 10 mL volumetric flask. Methanol was added to dissolve the sample, and the solution was diluted to volume to prepare a stock solution at a concentration of 1 mg/mL. The stock solution was subsequently diluted 20-fold with methanol containing 4 ppm 2-amino-3-(2-chlorophenyl)propionic acid to obtain a final QYG concentration of 50 μg/mL. The diluted solution was vortexed for 30 s, sonicated at room temperature for 15 min, and centrifuged at 12,000 rpm and 4 °C for 10 min. The resulting supernatant was filtered through a 0.22 μm membrane and transferred to an injection vial. Chromatographic separations were performed on an ACQUITY UPLC^®^ HSS T3 column (2.1 × 100 mm, 1.8 µm; Waters Corporation, Milford, MA, USA) maintained at 40 °C, with a flow rate of 0.3 mL/min and an injection volume of 2 μL. For the positive electrospray ionization mode (ESI (+)), the mobile phases were 0.1% formic acid in water (A2) and 0.1% formic acid in acetonitrile (B2) under the following gradient: 0–1 min, 8% B2; 1–8 min, 8–98% B2; 8–10 min, 98% B2; 10–10.1 min, 98–8% B2; 10.1–12 min, 8% B2. For the negative electrospray ionization mode (ESI (−)), the mobile phases were 5 mmol/L ammonium formate in water (A3) and acetonitrile (B3), following the same gradient. Mass spectrometric detection was conducted in Full MS-ddMS^2^ (data-dependent MS/MS) mode, with sheath gas at 40 arbitrary units, auxiliary gas at 10 arbitrary units, spray voltage at 3.50 kV (ESI (+)) and −2.50 kV (ESI (−)), capillary temperature at 325 °C, and an MS^1^ mass range of m/z 100–1000 at a resolving power of 60,000 FWHM. Up to four data-dependent MS^2^ scans (resolving power: 15,000 FWHM) were acquired per cycle, providing a comprehensive profile of the QYG extract.

### 2.2. Animal Modeling and Therapeutic Intervention

#### 2.2.1. Animals and Drug Administration

Female Kunming (KM) mice were used in two age cohorts: young adult mice aged 5 months and aged mice aged 11 months. The animals were purchased from the Hubei Provincial Center for Disease Control and Prevention (Wuhan, Hubei, China; certification No. SCXK (E) 2020-0018). All mice were housed under a 12 h light–dark cycle with free access to food and water, at a controlled temperature of 20–24 °C and relative humidity of 40–60%. Animal care and experimental procedures were conducted in accordance with the “Guidelines for the Care and Use of Experimental Animals” (Ministry of Science and Technology, 2010) and were approved by the Ethics Committee of Central South University for Nationalities (Approval No. 2023-scuec-034).

**Figure 1:**
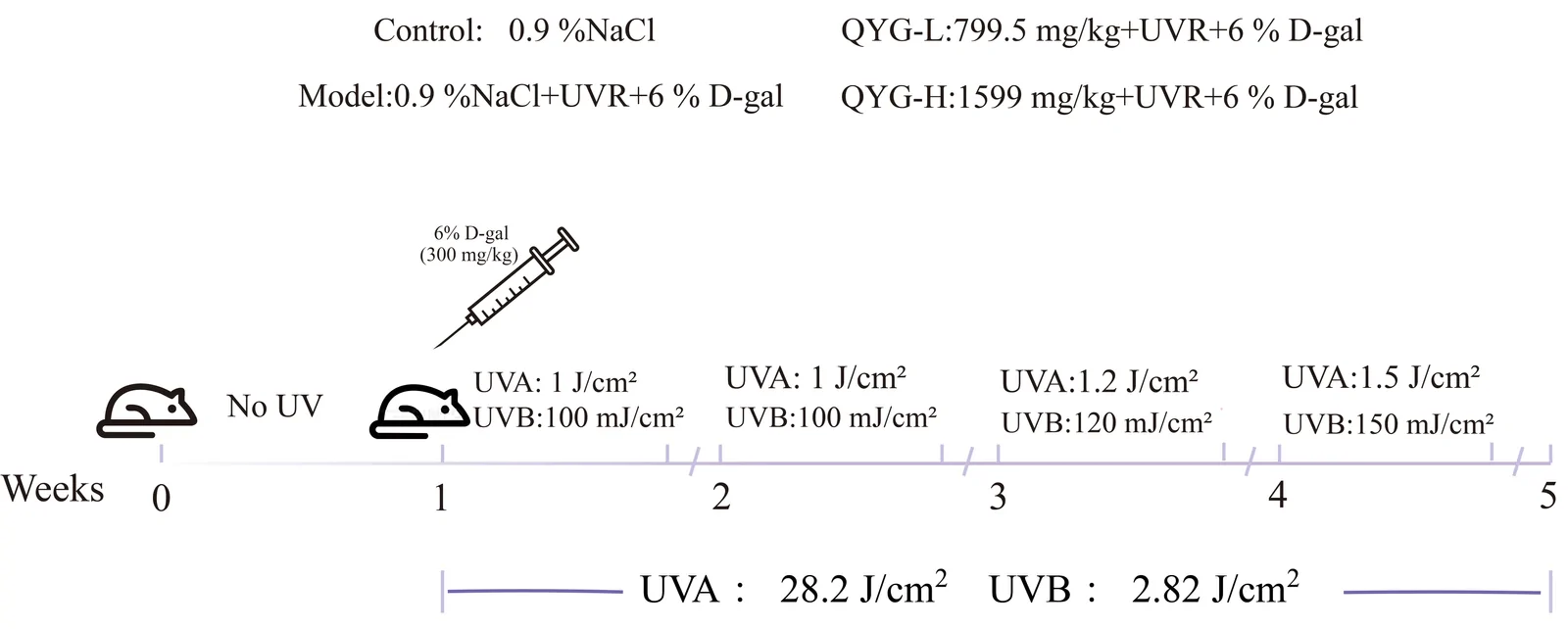
Experimental protocol for establishing the combined D-galactose and UVA/UVB-induced skin photoaging model and QYG gavage intervention in mice. Mice received daily subcutaneous injection of 6% D-galactose solution at 300 mg/kg and were exposed to UV light for six consecutive days followed by one day of rest each week for four weeks. The UVA irradiation dose was 1.0 J/cm^2^ during weeks 1–2, 1.2 J/cm^2^ in week 3, and 1.5 J/cm^2^ in week 4. The UVB irradiation dose was 100 mJ/cm^2^ during weeks 1–2, 120 mJ/cm^2^ in week 3, and 150 mJ/cm^2^ in week 4. The cumulative irradiation doses were 28.2 J/cm^2^ for UVA and 2.82 J/cm^2^ for UVB. Mice were divided into four groups: control (0.9% NaCl), model (0.9% NaCl + D-galactose + UVR), QYG low-dose group (799.5 mg/kg), and QYG high-dose group (1599 mg/kg), with QYG administered once daily by oral gavage.

After one week of acclimatization, the dorsal hair of all mice was shaved using an electric shaver, and residual hair was removed with depilatory cream (Veet, Reckitt Benckiser, Group plc, Slough, UK) to expose an area of approximately 3 × 4 cm^2^ (Figure 1). Mice then received a daily subcutaneous injection of 6% D-galactose solution at a dose of 300 mg/kg at the nape of the neck. Mice were exposed to UVA and UVB irradiation in a custom-designed UV irradiation chamber equipped with three UVA lamps (Actinic BL TL-D 18W/10, Philips/Signify, Eindhoven, The Netherlands) and two UVB lamps (G8T5E, Sankyo Denki Co., Ltd., Hiratsuka, Kanagawa, Japan). Irradiance was measured using a UV radiometer (ST-513, Sentry Optronics Corp., New Taipei City, Taiwan, China). Dorsal skinfold thickness was measured using a digital caliper (DHGDYKC1504, DELIXI, Hangzhou, China). During weeks 1 and 2, the irradiation doses were set at 1.0 J/cm^2^ for UVA and 100 mJ/cm^2^ for UVB. In week 3, the doses were increased to 1.2 J/cm^2^ for UVA and 120 mJ/cm^2^ for UVB. In week 4, the doses were further increased to 1.5 J/cm^2^ for UVA and 150 mJ/cm^2^ for UVB. Irradiation was performed for six consecutive days followed by one day of rest each week. The cumulative irradiation doses were 28.2 J/cm^2^ for UVA and 2.82 J/cm^2^ for UVB.

QYG extract was freshly dissolved in sterile 0.9% saline and administered once daily by oral gavage throughout the experimental period. Young mice were randomly assigned to five experimental groups (*n* = 6 per group): Blank (0.9% saline), Model (0.9% saline + UVR + 6% D-gal), VC (100 mg/kg/day vitamin C + UVR + 6% D-gal), QYG-L (799.5 mg/kg/day QYG + UVR + 6% D-gal), and QYG-H (1599 mg/kg/day QYG + UVR + 6% D-gal). Aged mice were randomly assigned to four experimental groups (*n* = 6 per group): Blank (0.9% saline), Model (0.9% saline + UVR + 6% D-gal), QYG-L (799.5 mg/kg/day QYG + UVR + 6% D-gal), and QYG-H (1599 mg/kg/day QYG + UVR + 6% D-gal). The QYG-L dose was calculated from the traditional adult daily dose using body surface area conversion and adjusted according to the extraction yield, while QYG-H was set as twice the equivalent dose to evaluate dose-dependent effects. The experimental design and irradiation protocol are shown in Figure 1. Skin condition was scored every three days, and dorsal skinfold thickness was measured every two days using a digital caliper by gently pinching the dorsal skin at the midpoint between the neck and hind limbs. At the end of the experiment, all mice were euthanized by CO_2_ inhalation according to the approved animal protocol, and death was confirmed before dorsal skin samples were collected. The collected skin tissues were rinsed with sterile saline and used for histological and molecular analyses.

#### 2.2.2. Skin Wrinkle Scoring and Thickness Measurement

Mice were monitored daily for erythema, blisters, or ulceration (irradiation terminated if observed). Skin aging phenotypes (thickening, laxity, wrinkles) were graded using the Kong and Bisset scale (Table 1): 0 (no wrinkles) to 6 (severe wrinkles with lesions) [20]. Skinfold thickness was measured every two days for 4 weeks by gently pinching the dorsal midpoint between the neck and hind limbs using a digital caliper.

#### 2.2.3. Biochemical Analysis

Approximately 0.1 g of dorsal skin tissue was weighed, cut into small pieces, and transferred to a precooled grinding tube containing 900 μL of ice-cold 0.9% physiological saline, corresponding to a tissue-to-buffer ratio of 1:9 (*w*/*v*) and yielding a 10% tissue homogenate. Three to four stainless-steel grinding beads with a diameter of 3 mm were added to each tube. The samples were homogenized using a tissue grinder (HY-21Y, Tianjin Ounuo, Tianjin, China) operated at −20 °C for six cycles, with each cycle consisting of 60 s of homogenization followed by a 30 s pause. The homogenates were centrifuged at 12,000× *g* for 15 min at 4 °C. The supernatants were transferred to clean 1.5 mL microcentrifuge tubes and centrifuged again at 12,000× *g* and 4 °C for 15 min. The final supernatants were collected and stored at −20 °C until analysis.

The total protein concentrations in the skin tissue supernatants were determined using the Coomassie Protein Assay Kit based on Coomassie Brilliant Blue G-250 (Thermo Scientific, Waltham, MA, USA; Cat. No. 23200), with absorbance measured at 595 nm using a multimode microplate reader (Synergy HTX, BioTek Instruments, Winooski, VT, USA). Superoxide dismutase (SOD) activity was measured using a WST-1 assay kit (Cat. No. A001-3), glutathione peroxidase (GSH-Px) activity using Cat. No. A005-1, malondialdehyde (MDA) content using a thiobarbituric acid assay kit (Cat. No. A003-1), catalase (CAT) activity using Cat. No. A007-2-1, and reactive oxygen species (ROS) levels using Cat. No. E004-1-1. All kits were obtained from Nanjing Jiancheng Bioengineering Institute (Nanjing, China), and the assays were performed using the final skin tissue supernatants according to the manufacturer’s instructions. The activities of SOD, GSH-Px, and CAT and the MDA content were normalized to the total protein concentration. ROS levels were determined based on fluorescence intensity.

Hyaluronic acid (HA) concentrations in the skin tissue supernatants were determined using a mouse HA enzyme-linked immunosorbent assay (ELISA) kit (Bioswamp, Wuhan, China; Cat. No. MU30353) according to the manufacturer’s instructions.

For serum preparation, whole blood samples were allowed to clot at room temperature for 1 h and then centrifuged at 2000× *g* for 15 min. The resulting serum was collected for ELISA analysis. Serum interleukin-6 (IL-6), matrix metalloproteinase-3 (MMP-3), and tumor necrosis factor-α (TNF-α) concentrations were measured using mouse ELISA kits from Bioswamp (Wuhan, China), with catalog numbers MU30044-96, MU30434-96T, and MU30030-96T, respectively, according to the manufacturer’s instructions.

#### 2.2.4. RT-PCR

Total RNA was isolated from mouse skin tissues using the ABclone RNA extraction kit (Cat# RK30120), followed by DNase I treatment to eliminate genomic DNA. cDNA synthesis was performed with 2 μg RNA using the Thermo Fisher Reverse Transcription Kit (Cat# K1691, including gDNA removal). For qPCR, reactions (20 μL) contained 10 μL 2× SYBR Green Master Mix, 0.5 μL forward/reverse primers (10 μM), and 2 μL cDNA template, and were analyzed on a QuantStudio 5 system (Applied Biosystems, Thermo Fisher Scientific, Waltham, MA, USA) under the following conditions: 95 °C for 10 min; 40 cycles of 95 °C for 15 s; and 60 °C for 30 s. Relative gene expression was normalized to β-actin via the 2^−ΔΔCt^ method (primer sequences provided in Supplementary Table S2).

#### 2.2.5. Histological Analysis

The skin tissue was fixed in 4% paraformaldehyde for 24 h and was embedded with paraffin after gradient-alcohol dehydration. Then, 3 µm-thick sections were used in Hematoxylin–eosin (H&E), Victoria blue staining, and Masson’s trichrome staining. All sections were scanned using a Pannoramic 250 Flash III slide scanner (3DHistech, Ltd., Budapest, Hungary), and images were acquired using CaseViewer software v2.4.0.119028 (3DHistech, Ltd., Budapest, Hungary).

### 2.3. Cells

#### 2.3.1. Isolation and Culture of Primary Mouse Dermal Fibroblasts (MDFs)

Primary mouse dermal fibroblasts were isolated from female C57BL/6 mice. Mice were deeply anesthetized with isoflurane inhalation, and deep anesthesia was confirmed by the absence of pedal reflex. The mice were then euthanized by CO_2_ inhalation according to the approved animal protocol. Death was confirmed before tissue collection. Dorsal skin was disinfected with 75% ethanol and aseptically excised into ice-cold PBS containing penicillin–streptomycin–amphotericin B. Subcutaneous fat and blood vessels were removed, and the tissue was minced into approximately 3 mm^2^ fragments for overnight digestion in Dispase II (2.5 mg/mL PBS) at 4 °C. The next day, the epidermis was separated from the dermis, and dermal fragments were mechanically dissociated with 0.25% trypsin-EDTA using a glass homogenizer. Digestion was terminated with F12 medium containing 10% FBS. Cell suspensions were filtered through a 100 μm cell strainer, centrifuged at 1000 rpm for 5 min, and cultured in complete F12 medium supplemented with 10% FBS, B-27, ITS-G, GlutaMAX, and antibiotics at 37 °C with 5% CO_2_. Cells between passages 3 and 5 were used for subsequent experiments at 80–90% confluence.

#### 2.3.2. Cell Modeling

The UVB irradiation system consisted of UVB lamps (G8T5E, Sankyo Denki Co., Ltd., Hiratsuka, Kanagawa, Japan) mounted above a Class II type A2 biosafety cabinet (BSC-1304IIA2, Suzhou Antai Airtech Co., Ltd., Suzhou, China). UVB irradiance was monitored using an ultraviolet radiometer (ST-513, Sentry Optronics Corp., New Taipei City, Taiwan, China). Before irradiation, the cabinet was sterilized by UV exposure for 30 min, and the UVB lamps were pre-warmed and stabilized. The radiation intensity was calibrated to 7500 μW/cm^2^ at a distance of 8 cm between the lamp and the culture plate using a radiometer. Logarithmic-phase primary mouse dermal fibroblasts were seeded into 96-well plates at 10,000 cells/well and cultured for 24 h until reaching 80–90% confluence. Before irradiation, the culture medium was replaced with sterile PBS to cover the cell monolayer, and cells were exposed to UVB at doses of 6, 7.5, 9, and 10.5 J/cm^2^. After irradiation, PBS was replaced with complete medium containing 10% FBS, and cells were cultured for another 24 h before cell viability assessment using the CCK-8 assay. Absorbance was measured at 450 nm using a multimode microplate reader (Synergy HTX, BioTek Instruments, Winooski, VT, USA), and data were analyzed using GraphPad Prism 9.

#### 2.3.3. Drug-Containing Serum Treatment

For the preparation of drug-containing serum, SD rats were orally administered QYG at a dose of 60.45 mg/rat/day for five consecutive days. After the final administration, rats were deeply anesthetized with isoflurane, and deep anesthesia was confirmed by the absence of pedal reflex. Blood was collected from the abdominal aorta under deep anesthesia as a terminal procedure, with no recovery allowed. Immediately after blood collection, animals were euthanized by CO_2_ inhalation according to the approved animal protocol, and death was confirmed before carcass disposal. Control serum was obtained from untreated rats using the same procedure. The collected blood was centrifuged to isolate serum, which was subsequently inactivated at 56 °C for 30 min and filtered through a 0.22 μm membrane for sterilization. The drug-containing serum was then mixed with blank serum to obtain working concentrations of 5%, 7.5%, 10%, 12.5%, and 15%.

#### 2.3.4. Western Blotting

Mouse dermal fibroblasts (MDFs) were extracted using RIPA lysis buffer, which was supplemented with protease inhibitors (Beijing Solarbio Science & Technology Co., Ltd., Beijing, China). Subsequently, the sample protein content was measured using a BCA assay kit and subjected to sodium dodecyl sulfate-polyacrylamide gel electrophoresis (SDS-PAGE). The samples were transferred onto nitrocellulose (NC) transfer membranes (LABSELECT, Beijing, China) and blocked at room temperature for 1 h using 5% skim milk. The primary antibodies anti-Phospho-AMPKα (1:1000, Cell signaling Technology, 2535T), anti-AMPKα (1:2000, Proteintech, 10929-2-AP), anti-Phospho-ACC1 (1:500, Proteintech, 29119-1-AP), and anti-PGC-1α (1:5000, Proteintech, 66369-1-Ig) were added and incubated at 4 °C overnight. β-actin served as the internal control. After incubating the membrane with the fluorescent secondary antibody for 1 h at room temperature, the bands were scanned using an Odyssey DLx Imaging System (LI-COR Biosciences, Lincoln, NE, USA) and analyzed with the built-in software Empiria Studio v2.2.0.141 (LI-COR Biosciences, Lincoln, NE, USA).

#### 2.3.5. Evaluation of Mitochondrial Membrane Potential

The JC-1 mitochondrial membrane potential detection kit (G1515, Servicebio, Wuhan, China) was used to evaluate the changes in mitochondrial membrane potential following the manufacturer’s instructions. Briefly, cells were washed with PBS, followed by immersion in a JC-1 dye solution at 37 °C for 30 min. After the incubation, cells underwent two additional washes with a dilution buffer, and images were recorded using a laser scanning confocal microscope (Leica Microsystems, Wetzlar, Germany). The green or red fluorescence intensity was analyzed by ImageJ v1.54.

#### 2.3.6. ROS Detection

Intracellular reactive oxygen species (ROS) levels were measured using a 2′,7′-dichlorodihydrofluorescein diacetate (DCFH-DA) probe (Cat. No. E004-1-1, Nanjing Jiancheng Bioengineering Institute, Nanjing, China). Cells were incubated with 10 μM DCFH-DA at 37 °C for 30 min in the dark. ROS fluorescence was subsequently evaluated by confocal fluorescence microscopy, microplate-based fluorescence measurement, and flow cytometry. Fluorescence images were acquired using a STELLARIS 5 laser scanning confocal microscope equipped with a DMi8 fully motorized inverted fluorescence microscope (Leica Microsystems, Wetzlar, Germany), and fluorescence intensity was quantified using ImageJ software. Microplate-based fluorescence intensity was measured at excitation/emission wavelengths of 488/525 nm using a Synergy HTX multimode microplate reader (BioTek Instruments, Winooski, VT, USA). Flow cytometric analysis was performed using a BD Accuri C6 flow cytometer (BD Biosciences, San Jose, CA, USA), and the resulting data were analyzed using FlowJo software v10.8.1.

#### 2.3.7. Senescence-Associated β-Galactosidase (SA-β-Gal) Staining Assay

Cellular senescence was detected using a SA-β-gal staining kit (Beyotime Biotechnology, Shanghai, China) according to the manufacturer’s protocol. Briefly, cells were fixed, incubated with SA-β-gal staining solution overnight at 37 °C, and observed under an IX73 inverted microscope (Olympus Corporation, Tokyo, Japan). Senescent cells were identified by blue cytoplasmic staining, and the percentage of SA-β-gal-positive cells was quantified from five randomly selected fields per well.

### 2.4. Statistical Analysis

Data are expressed as mean ± SEM. Statistical analyses were performed using SPSS 20.0. One-way ANOVA was used for comparisons among groups, with *p* < 0.05 considered statistically significant. Graphs were generated using GraphPad Prism 9.0. Data normality was assessed using the Shapiro–Wilk test, and homogeneity of variances was evaluated using Levene’s test. For data satisfying the assumptions of normality and homogeneity of variances, comparisons among multiple groups were performed using one-way analysis of variance (ANOVA), followed by Tukey’s honestly significant difference post hoc test. When the assumption of homogeneity of variances was violated, Welch’s ANOVA followed by the Games–Howell post hoc test was used. A value of *p* < 0.05 was considered statistically significant.

## 3. Results

### 3.1. QYG Attenuates UV-Induced Skin Photoaging in Young and Aged Mice

**Figure 2:**
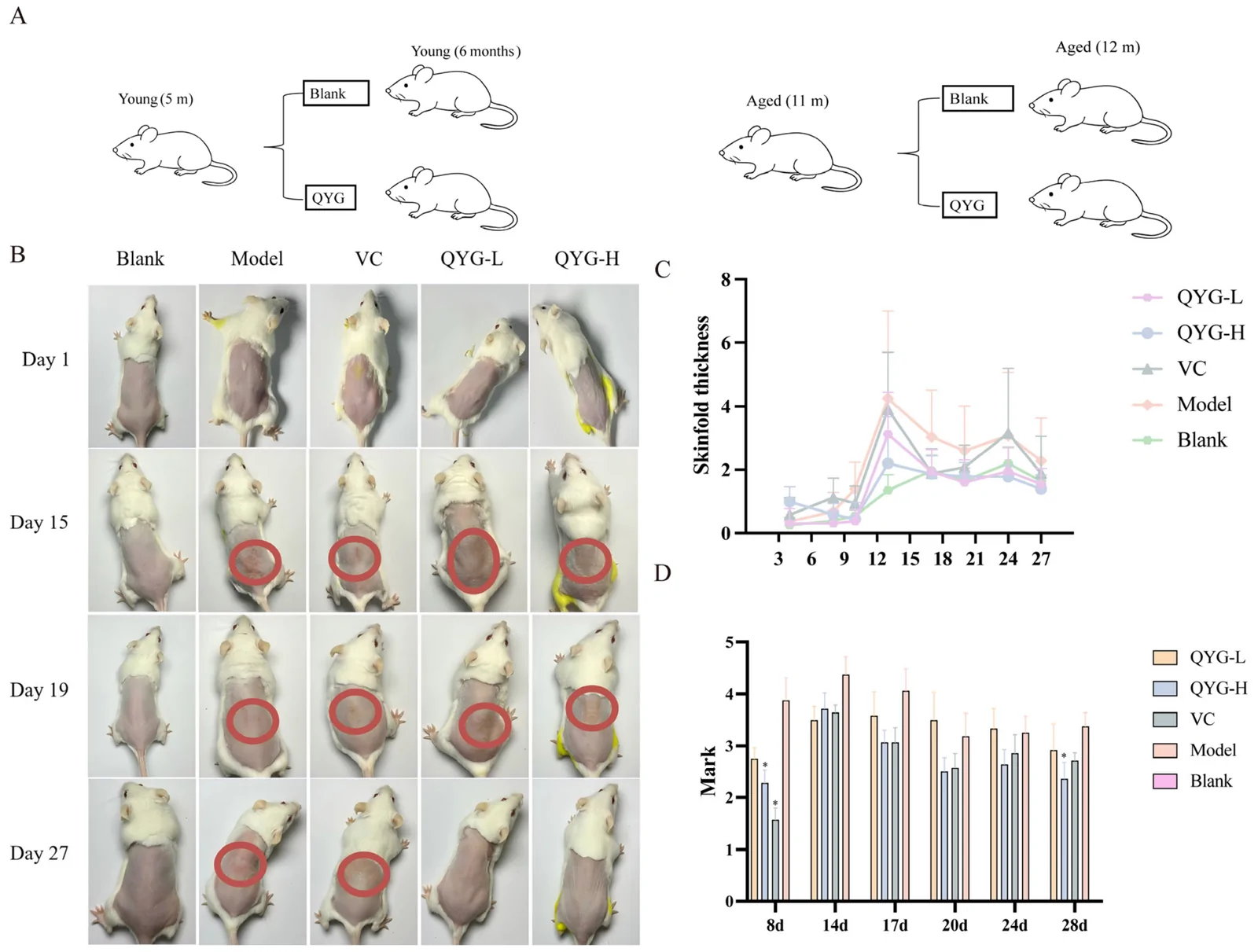
Experimental design and effects of QYG on UV-induced skin photoaging in young mice. (**A**) Experimental design showing the 1-month interventions initiated in young adult (5-month-old) and aged (11-month-old) Kunming (KM) mice. (**B**) Representative dorsal skin appearance of young mice during the intervention; red circles indicate areas of wrinkles, erythema, and scaling. (**C**) Dynamic changes in dorsal skinfold thickness (*n* = 6 per group). (**D**) Skin condition scores during the intervention (*n* = 6 per group). VC, vitamin C; QYG-L, low-dose QYG; QYG-H, high-dose QYG. Data are presented as the mean ± SEM. Statistical significance is indicated as * *p* < 0.05.

In this study, photoaging models were established in both young (5-month-old) and aged (11-month-old) Kunming mice through exposure to ultraviolet (UVA + UVB), followed by QYG administration for one month to evaluate its anti-photoaging effects (Figure 2A).

**Figure 3:**
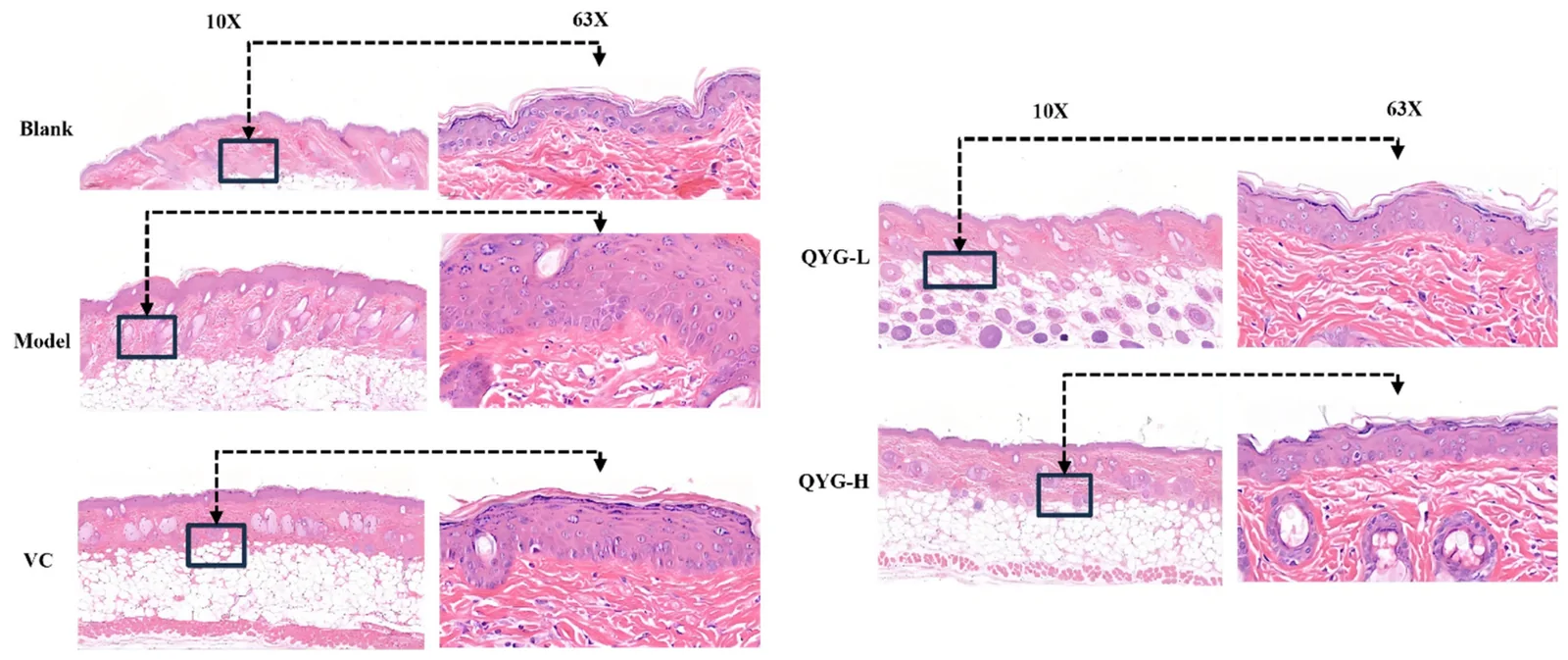
Representative hematoxylin and eosin (H&E)-stained dorsal skin sections from young mice at 10× and 63× magnification. Scale bars: 100 μm for 10× images and 20 μm for 63× images.

In 5-month-old mice, QYG markedly improved skin appearance by reducing erythema, scaling, and wrinkles (Figure 2B). QYG also inhibited the increase in dorsal skinfold thickness (Figure 2C). After 28 days, the skin condition score in the high-dose QYG group was approximately 33% lower than that in the Model group (Figure 2D). H&E staining further showed reduced epidermal thickening and improved dermal structural organization after QYG treatment (Figure 3). Molecular and biochemical assays showed that QYG downregulated inflammatory factors IL-6, IL-17, and TNF-α and matrix degradation-related genes MMP-3, MMP-9, and PAR1, and suppressed apoptosis-related genes Caspase-3 and Caspase-9 (Supplementary Figure S1). In terms of oxidative stress, high-dose QYG significantly increased SOD activity by about 1.2-fold and GSH-Px by about 40%, while reducing MDA levels by about 50% (Supplementary Figure S1). In addition, QYG raised hyaluronic acid (HA) levels by about 2.4-fold, contributing to skin hydration and elasticity. Masson staining further confirmed that QYG improved collagen fiber content and structural integrity (Supplementary Figure S1).

**Figure 4:**
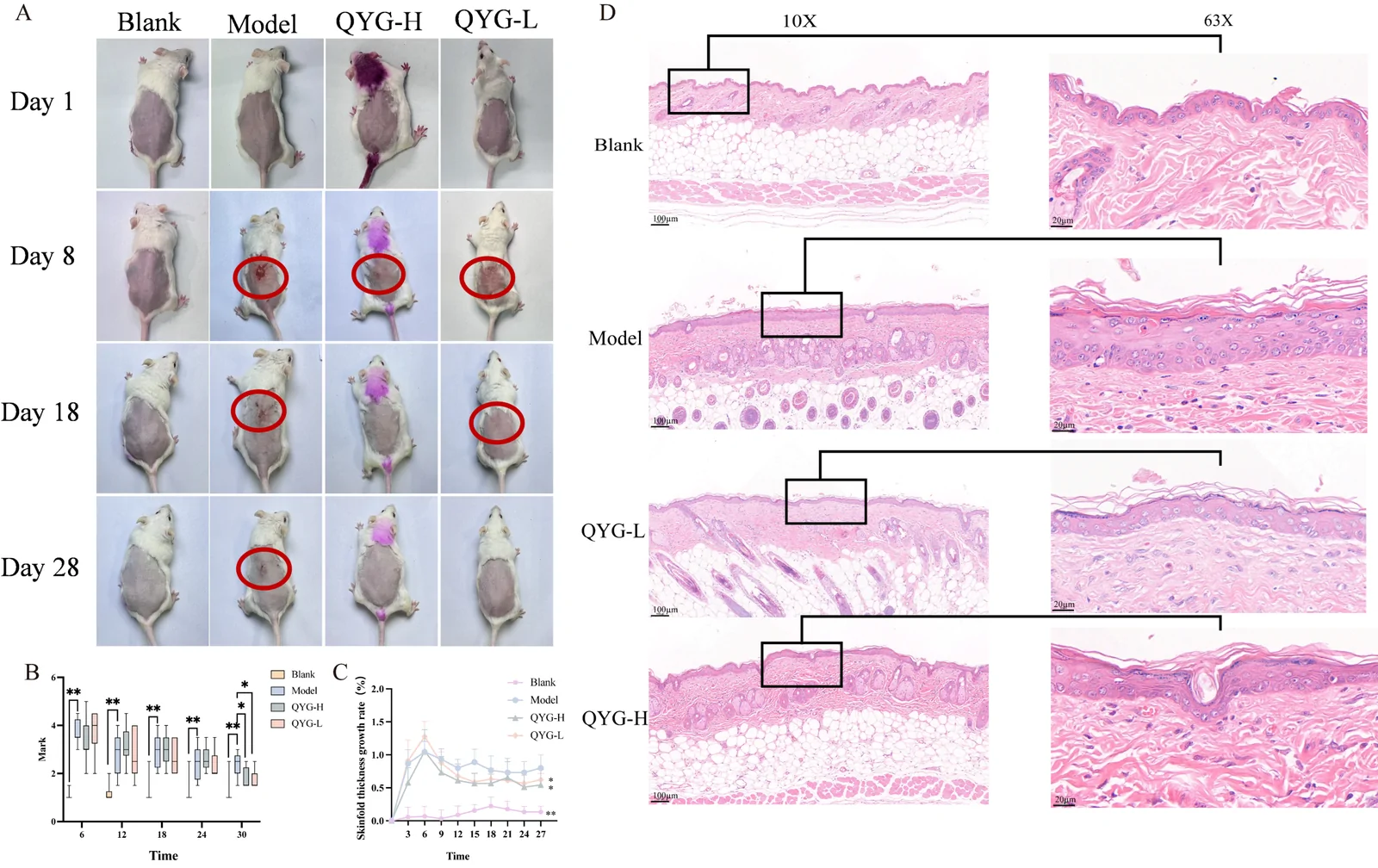
QYG attenuates UV-induced skin photoaging phenotypes and histological alterations in aged mice. (**A**) Representative dorsal skin appearance of aged mice during the intervention; red circles indicate areas of wrinkles, erythema, and scaling. (**B**) Skin condition scores during the intervention (*n* = 6 per group). (**C**) Growth rate of dorsal skinfold thickness during the intervention (*n* = 6 per group). (**D**) Representative H&E-stained dorsal skin sections at 10× and 63× magnification. Scale bars: 100 μm for 10× images and 20 μm for 63× images. Statistical significance is indicated as * *p* < 0.05 and ** *p* < 0.01.

**Figure 5:**
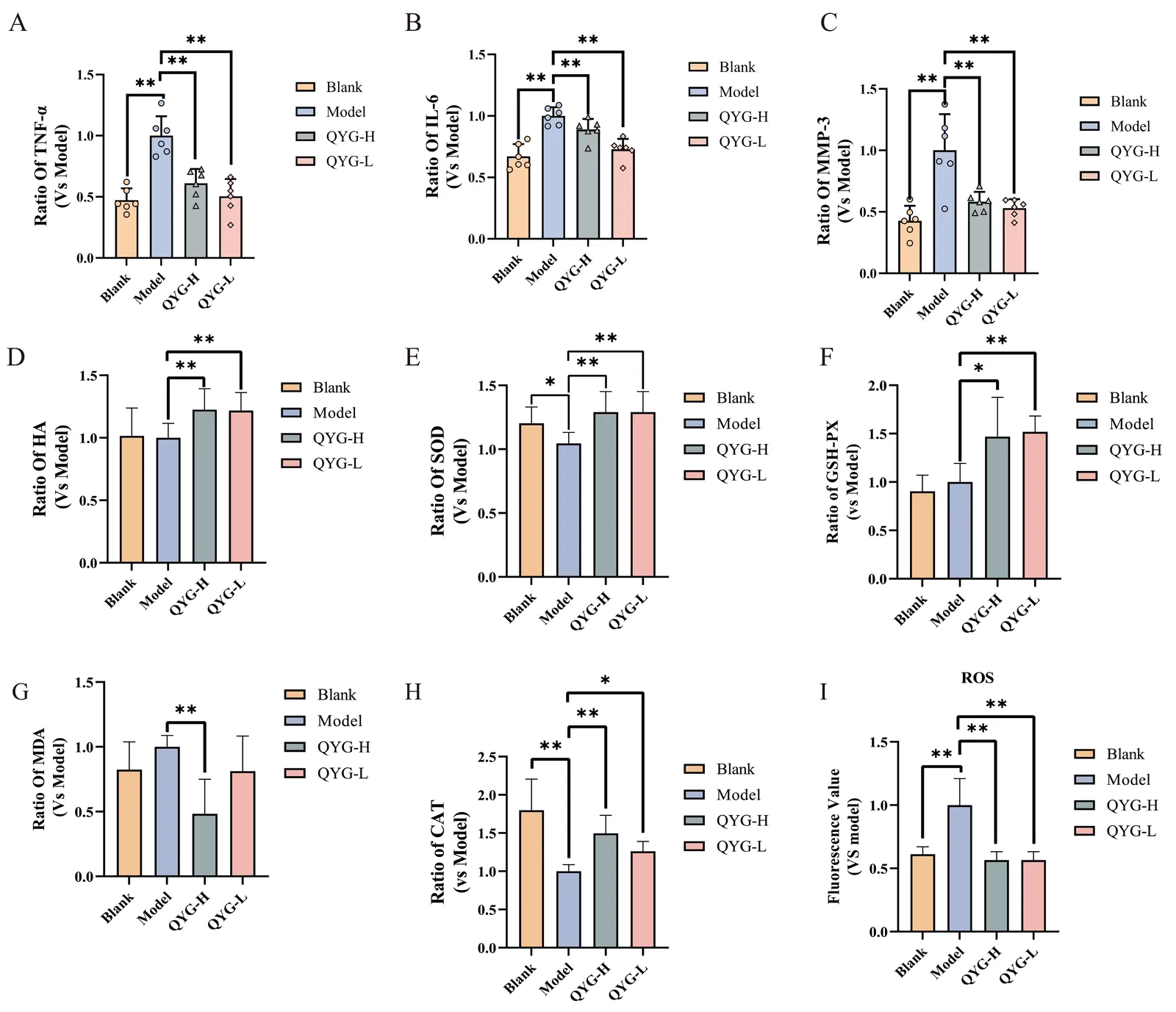
Effects of QYG on serum inflammatory mediators and skin biochemical indices in aged mice. (**A**–**C**) Serum levels of tumor necrosis factor-α (TNF-α), interleukin-6 (IL-6), and matrix metalloproteinase-3 (MMP-3), respectively, measured by enzyme-linked immunosorbent assay. (**D**–**I**) Skin levels of hyaluronic acid (HA), superoxide dismutase (SOD), glutathione peroxidase (GSH-Px), malondialdehyde (MDA), catalase (CAT), and reactive oxygen species (ROS), respectively. ROS levels were determined based on fluorescence intensity. Data are presented as the mean ± SEM (*n* = 6 per group). Statistical significance is indicated as * *p* < 0.05 and ** *p* < 0.01.

To determine whether QYG retained protective effects in aged animals, the intervention was further evaluated in 11-month-old mice. The corresponding skin phenotypes and histological results are presented in Figure 4, whereas serum inflammatory mediators and skin biochemical indices are presented in Figure 5. The model group developed severe skin damage, including erythema, roughness, and thickening. Compared with the model group, QYG significantly improved these symptoms, lowering the skin score by about 30% (Figure 4B) and inhibiting epidermal thickening (Figure 4C), and also demonstrated robust anti-photoaging effects. Histological analysis revealed reduced epidermal thickness and more compact collagen arrangement in the QYG groups (Figure 4D).

Further mechanistic analysis showed that QYG significantly alleviated UV-induced systemic inflammation at the serum level. In the high-dose group, TNF-α and IL-6 concentrations decreased by about 50–60%, and MMP-3 levels fell to about 0.6-fold of the model group (Figure 5A–C), indicating effective suppression of inflammation and collagen degradation.

In the skin tissue, QYG also exhibited clear antioxidant and barrier-protective effects. In the high-dose group, HA content increased to about 1.3-fold of the model group (Figure 5D). SOD and GSH-Px activities were increased following QYG treatment (Figure 5E,F), while CAT activity increased by up to approximately twofold (Figure 5H). MDA and ROS levels decreased to approximately 0.6-fold and 0.5-fold of those in the Model group, respectively (Figure 5G,I).

Together, these results indicate that oral QYG alleviated UV-induced skin photoaging phenotypes in both young and aged mice.

### 3.2. Transcriptomic Analysis Reveals QYG Modulates Mitochondrial Energy Metabolism-Related Pathways

**Figure 6:**
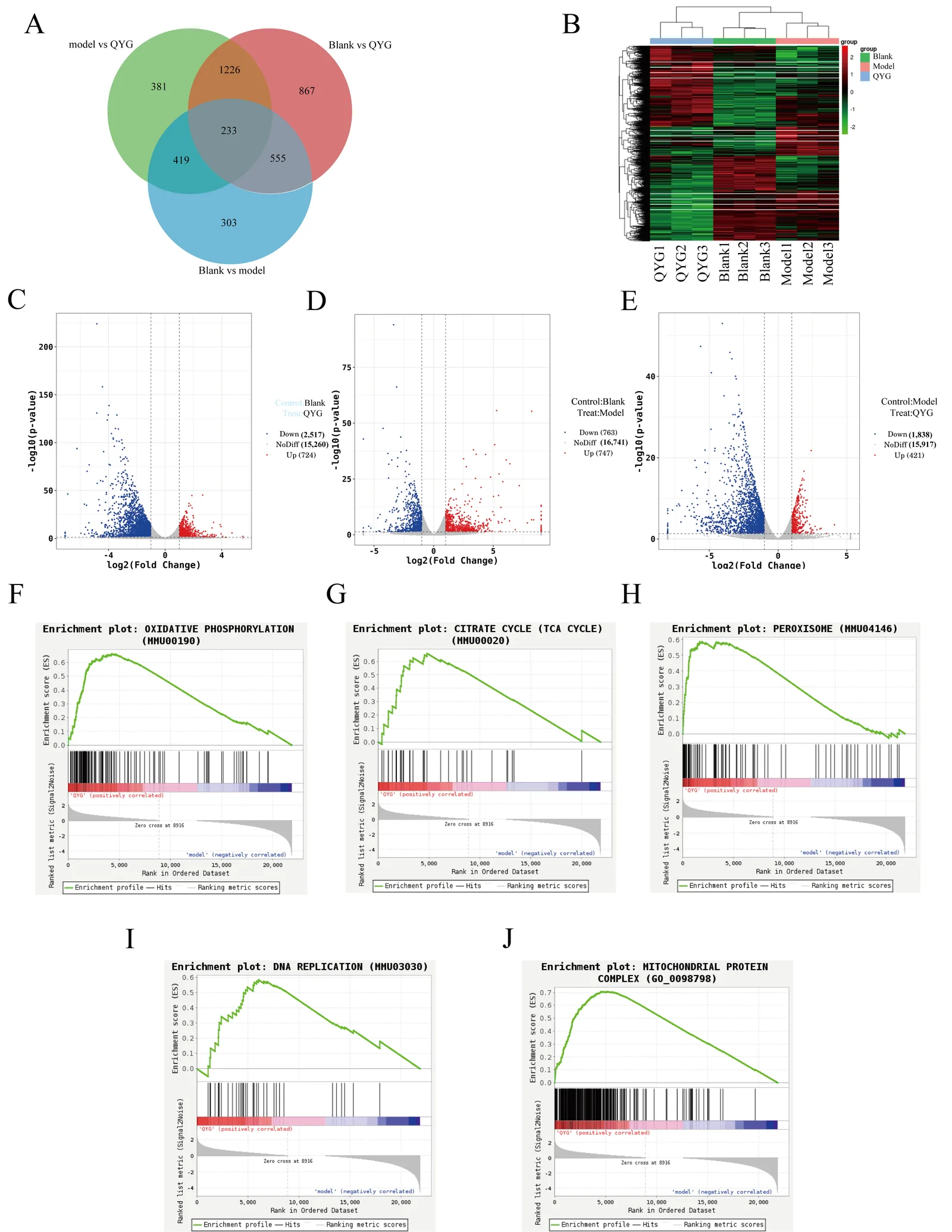
Transcriptomic analysis reveals that QYG modulates mitochondrial energy metabolism-related pathways in aged photoaged mice. Skin tissues from aged mice were subjected to RNA-seq analysis (*n* = 3 per group). (**A**) Venn diagram showing shared and comparison-specific differentially expressed genes (DEGs) among the Blank versus QYG, Blank versus Model, and Model versus QYG comparisons. (**B**) Hierarchical clustering heatmap showing the expression patterns of DEGs among the Blank, Model, and QYG groups. (**C**–**E**) Volcano plots showing significantly upregulated and downregulated genes in the comparisons of Blank versus QYG (**C**), Blank versus Model (**D**), and Model versus QYG (**E**). Red dots indicate upregulated genes, blue dots indicate downregulated genes, and gray dots indicate non-significant genes. (**F**–**J**) Gene Set Enrichment Analysis (GSEA) showing enrichment of oxidative phosphorylation (**F**), tricarboxylic acid (TCA) cycle (**G**), peroxisome (**H**), DNA replication (**I**), and mitochondrial protein complex (**J**). Green lines represent enrichment scores, and black vertical bars indicate the distribution of core enriched genes.

To further elucidate the protective mechanisms of QYG in the aged mouse model of UV-induced skin photoaging, transcriptomic profiling (RNA-seq) was performed on skin tissues. Differential expression analysis revealed distinct gene expression changes among the Blank, Model, and QYG groups (Figure 6A). Hierarchical clustering showed that the QYG group displayed a transcriptional profile clearly different from that of the Model group (Figure 6B).

The Venn diagram showed both shared and comparison-specific differentially expressed genes (DEGs) among the three pairwise comparisons (Figure 6A). In the QYG versus Model comparison, a total of 2259 DEGs were identified, including 421 upregulated and 1838 downregulated genes in the QYG group relative to the Model group (Figure 6E). Among these, 381 DEGs were specific to the QYG versus Model comparison, whereas 233 DEGs were shared among all three comparisons. Volcano plots further showed the distribution of upregulated and downregulated genes in the Blank versus QYG, Blank versus Model, and Model versus QYG comparisons (Figure 6C–E). GSEA revealed enrichment of oxidative phosphorylation, TCA cycle, peroxisome, DNA replication, and mitochondrial protein complex pathways, with leading-edge genes including the *Atp5* family, *Cox* family, *Aco2*, *Idh3a*, and *Cs* (Figure 6F–J).

### 3.3. Gut Microbiota 16S rRNA Sequencing Analysis

Based on the preceding transcriptomic results showing the enrichment of mitochondrial energy metabolism-related pathways in skin tissue, we further examined whether oral QYG treatment was accompanied by gut microbiota remodeling in aged photoaged mice. Considering that aging and UVR exposure can influence gut microbial composition [21,22], 16S rRNA sequencing was performed to compare the intestinal microbiota among the Blank, Model, and QYG groups.

**Figure 7:**
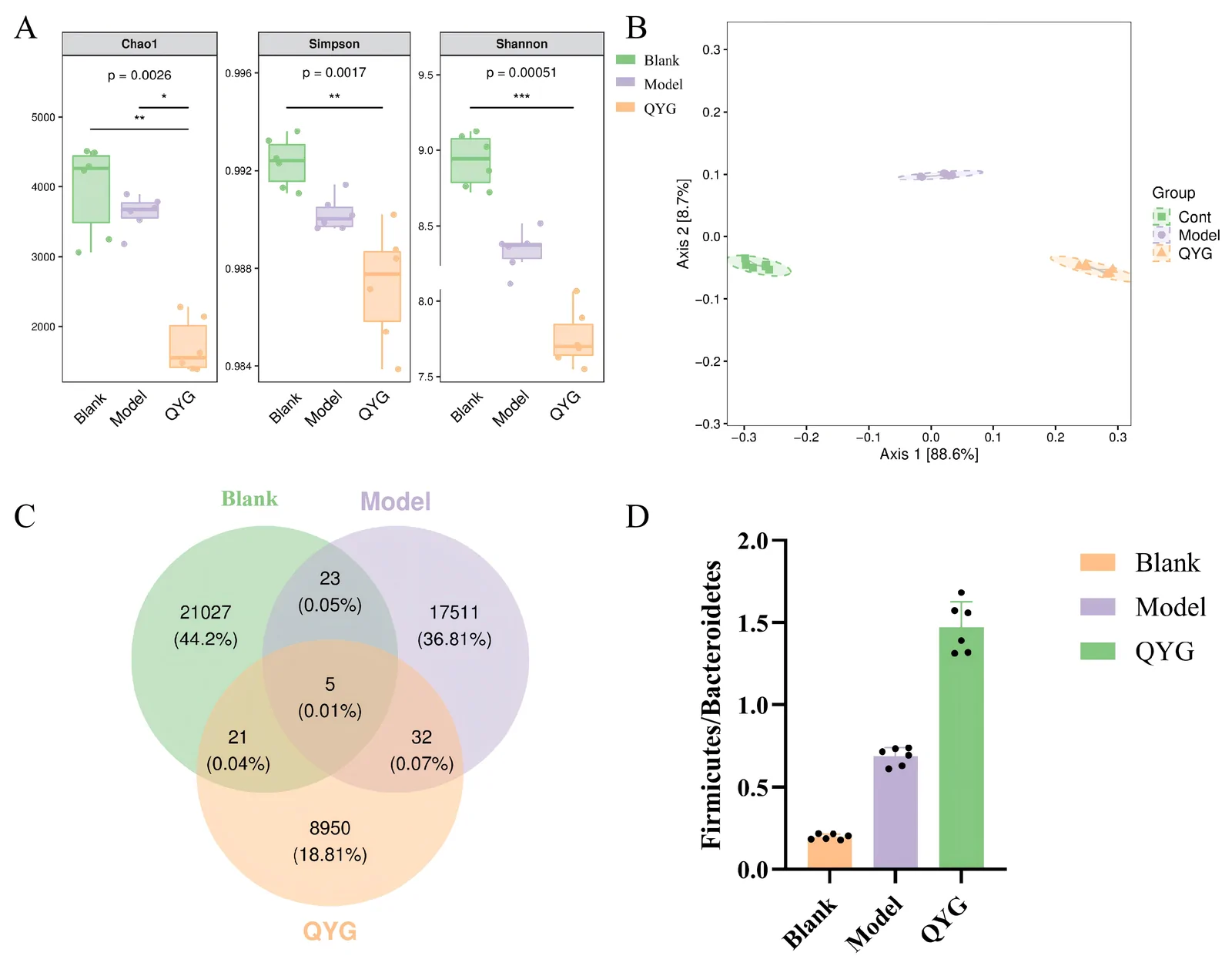
QYG reshaped gut microbial diversity and overall community structure in aged photoaged mice. (**A**) α-diversity indices, including Chao1, Simpson, and Shannon indices, among the Blank, Model, and QYG groups. (**B**) Principal coordinate analysis (PCoA) showing differences in overall gut microbial community structure among the three groups. (**C**) Venn diagram showing shared and group-specific amplicon sequence variants (ASVs). (**D**) Firmicutes/Bacteroidetes ratio among the three groups. Six biological replicates were included in each group (*n* = 6). Statistical significance is indicated as * *p* < 0.05, ** *p* < 0.01, and *** *p* < 0.001.

α-diversity analysis revealed significant differences among the three groups (Figure 7A). Compared with the Blank group, the Model group showed decreased Chao1, Simpson, and Shannon indices, indicating reduced microbial richness and diversity after photoaging induction. After QYG treatment, these indices were further decreased rather than restored to the Blank level, suggesting selective remodeling of the gut microbial community rather than a simple increase in overall diversity. This interpretation is consistent with recent views that α-diversity alone should not be regarded as a direct indicator of microbiome health, and that microbiota-targeted interventions may act through selective changes in community composition [23,24].

β-diversity analysis showed clear separation among the Blank, Model, and QYG groups, indicating distinct overall microbial community structures (Figure 7B). Consistently, the Venn diagram revealed marked differences in ASV distribution, with a large number of group-specific ASVs and only a small number of shared ASVs among all three groups (Figure 7C). At the phylum level, the Firmicutes/Bacteroidetes ratio was increased in the Model group compared with the Blank group and was further elevated after QYG treatment (Figure 7D). Because the F/B ratio alone cannot be interpreted as a simple indicator of microbial health or dysbiosis, subsequent taxonomic and functional analyses were performed to further characterize QYG-induced microbial remodeling.

**Figure 8:**
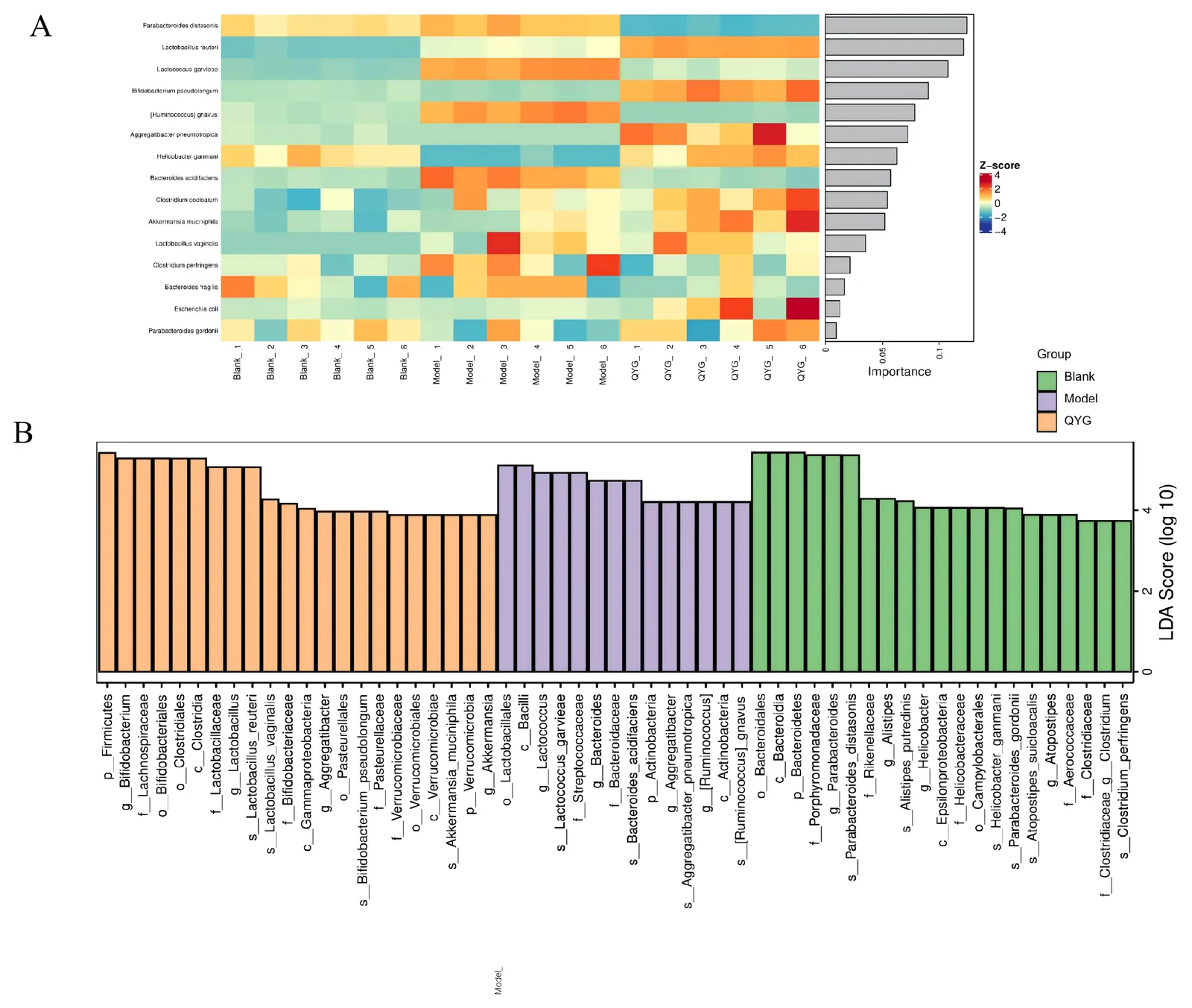
Random forest and LEfSe analyses identified discriminative gut microbial taxa among the Blank, Model, and QYG groups. (**A**) Random forest analysis showing discriminative bacterial species among the three groups. The heatmap shows the relative abundance of representative taxa across individual samples, and the right-hand bar plot indicates their variable importance. (**B**) Linear discriminant analysis effect size (LEfSe) LDA score plot showing differentially enriched taxa among the three groups. Green, purple, and orange indicate taxa enriched in the Blank, Model, and QYG groups, respectively. Six biological replicates were included in each group (*n* = 6).

Random forest analysis identified several discriminative bacterial species among the three groups, including *Parabacteroides distasonis*, *Lactobacillus reuteri*, *Lactococcus garvieae*, *Bifidobacterium pseudolongum*, *[Ruminococcus] gnavus*, *Akkermansia muciniphila*, *Bacteroides acidifaciens*, *Clostridium cocleatum*, *Escherichia coli*, and *Parabacteroides gordonii* (Figure 8A). LEfSe analysis further identified differentially enriched taxa among the three groups (Figure 8B). The QYG group was enriched in Firmicutes, Clostridia, Clostridiales, Lachnospiraceae, Lactobacillaceae, *Lactobacillus*, *Lactobacillus reuteri*, *Lactobacillus vaginalis*, Bifidobacteriaceae, Bifidobacteriales, *Bifidobacterium*, *Bifidobacterium pseudolongum*, Verrucomicrobia, and *Akkermansia muciniphila*. In contrast, the Model group was characterized by enrichment of Lactobacillales, Bacilli, *Lactococcus*, *Lactococcus garvieae*, Streptococcaceae, *Bacteroides*, *Bacteroidaceae*, *Bacteroides acidifaciens*, Actinobacteria, *Aggregatibacter*, and *[Ruminococcus] gnavus*. The Blank group was mainly enriched in Bacteroidales, Bacteroidia, Porphyromonadaceae, *Parabacteroides distasonis*, Rikenellaceae, *Alistipes*, Helicobacteraceae, and Clostridiaceae-related taxa. Notably, *Lactobacillus* and *Bifidobacterium* are classical probiotic-associated genera, whereas *Akkermansia muciniphila* is widely regarded as a next-generation beneficial microbial candidate associated with gut barrier and metabolic regulation [3,25,26]. These results suggest that QYG selectively reshaped the gut microbiota by enriching several health-associated or probiotic-related taxa.

**Figure 9:**
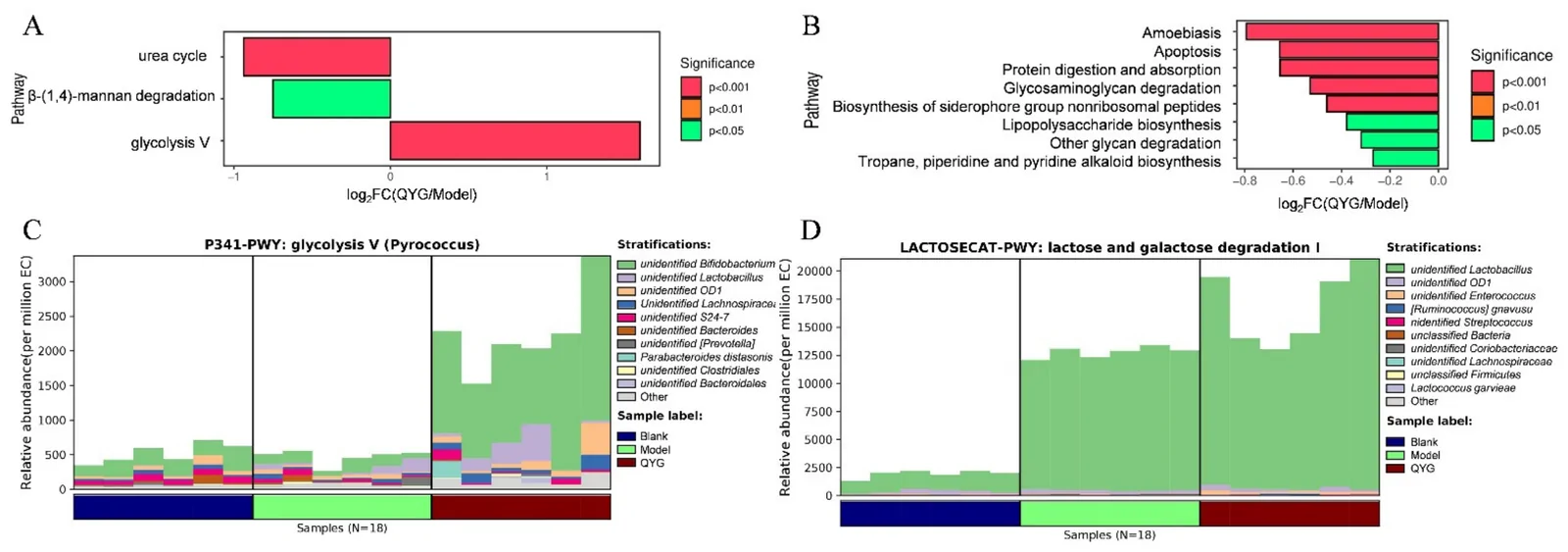
Predicted microbial metabolic functions altered by QYG treatment. (**A**) Differential MetaCyc pathways between the QYG and Model groups identified by metagenomeSeq analysis. Positive log_2_FC values indicate higher predicted pathway abundance in the QYG group, whereas negative values indicate lower predicted abundance in the QYG group. (**B**) Differential KEGG pathways between the QYG and Model groups identified by metagenomeSeq analysis. Bar colors indicate statistical significance. (**C**) Taxonomic stratification of the P341-PWY pathway, glycolysis V, generated by HUMAnN2 analysis. The stacked bars show the relative contribution of different bacterial taxa to this pathway across individual samples. (**D**) Taxonomic stratification of the LACTOSECAT-PWY pathway, lactose and galactose degradation I, generated by HUMAnN2 analysis. The stacked bars show the relative contribution of different bacterial taxa to this pathway across individual samples. Six biological replicates were included in each group (*n* = 6).

To determine whether QYG-induced taxonomic changes were accompanied by alterations in microbial metabolic potential, differential pathway analysis was performed using metagenomeSeq, followed by HUMAnN2-based pathway contribution analysis. In the metagenomeSeq analysis, log_2_FC was calculated as log_2_(QYG/Model), with positive values indicating higher predicted pathway abundance in the QYG group and negative values indicating lower abundance. MetaCyc pathway analysis showed that glycolysis V was significantly increased in the QYG group compared with the Model group, whereas the urea cycle and β-(1,4)-mannan degradation were decreased (Figure 9A). KEGG prediction further showed that several Model-associated microbial pathways, including protein digestion and absorption, glycosaminoglycan degradation, lipopolysaccharide biosynthesis, and glycan degradation, were decreased after QYG treatment (Figure 9B). These results indicate that QYG selectively regulated microbial metabolic pathways rather than uniformly enhancing all predicted microbial functions.

HUMAnN2-based taxonomic stratification was further used to identify microbial contributors to key differential pathways. P341-PWY: glycolysis V was markedly increased in the QYG group and was mainly contributed to by *Bifidobacterium-, Lactobacillus*-, OD1-, and Lachnospiraceae-associated taxa (Figure 9C), consistent with the LEfSe enrichment of *Bifidobacterium*- and *Lactobacillus*-related taxa. Given that dietary carbohydrates and plant-derived polysaccharides can directly interact with gut microbes and influence microbial metabolic outputs [3], the increased contribution of these taxa to glycolysis-related pathways may reflect altered microbial carbohydrate metabolism after QYG administration. LACTOSECAT-PWY: lactose and galactose degradation I was increased in the Model group compared with the Blank group and remained at a high level after QYG treatment (Figure 9D). Taxonomic stratification showed that this pathway was predominantly contributed to by *Lactobacillus*-associated taxa, indicating that QYG-induced microbial remodeling was accompanied by changes in Lactobacillus-associated carbohydrate utilization pathways.

### 3.4. Serum Metabolomics Analysis

The preceding transcriptomic analysis showed that QYG treatment significantly enriched mitochondrial energy metabolism-related pathways in skin tissue, including the TCA cycle and oxidative phosphorylation. Mitochondria are central hubs of cellular metabolism, supporting ATP production, redox balance, and biosynthetic precursor generation [27]. Functional prediction of the gut microbiota further indicated alterations in microbial glycolysis and carbohydrate utilization, suggesting that systemic metabolic remodeling may be involved in the anti-photoaging effects of QYG. Therefore, untargeted serum metabolomics was performed and integrated with chemical profiling and systemic exposure analysis of QYG constituents.

**Figure 10:**
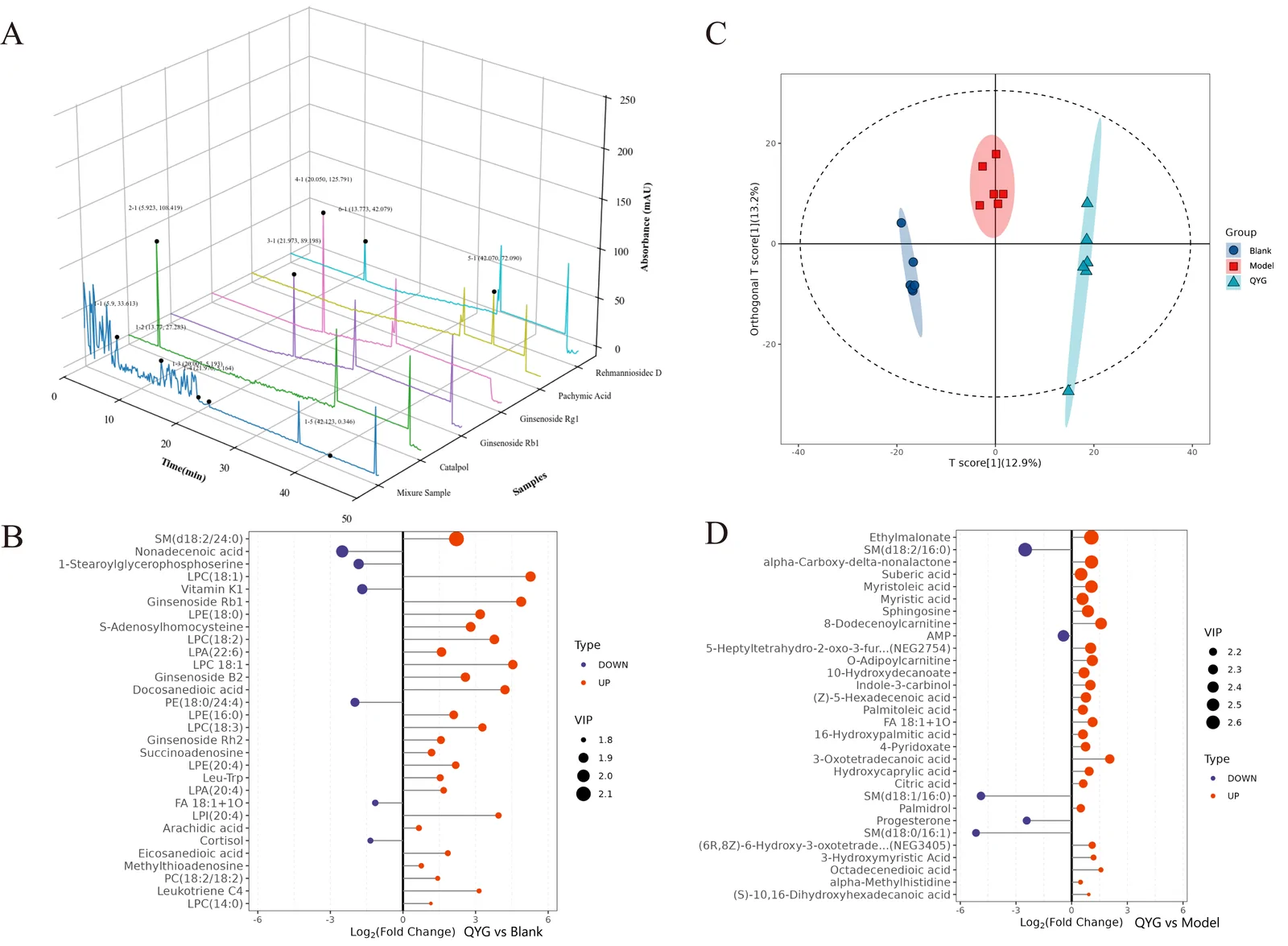
Chemical profiling of QYG and serum metabolomic analysis in aged mice with UV-induced photoaging. (**A**) HPLC waterfall chromatograms of representative reference standards and QYG samples, including catalpol, rehmannioside, ginsenoside Rg1, ginsenoside Rb1, and pachymic acid. (**B**) Lollipop plot showing representative differential serum metabolites between the QYG and Blank groups, including serum-exposed QYG-related constituents and other altered metabolites. (**C**) Orthogonal partial least-squares discriminant analysis (OPLS-DA) score plot showing metabolic separation among the Blank, Model, and QYG groups. (**D**) Lollipop plot showing representative differential serum metabolites between the QYG and Model groups. In panels (**B**,**D**), positive log_2_ fold-change values indicate metabolites increased in the QYG group, whereas negative values indicate metabolites decreased in the QYG group. Dot colors indicate upregulated or downregulated metabolites, and dot size represents variable importance in projection (VIP) values. Serum samples from six biological replicates per group were used for metabolomic analysis (*n* = 6).

HPLC analysis detected five representative marker compounds selected according to the Chinese Pharmacopoeia, including catalpol, rehmannioside, ginsenoside Rg1, ginsenoside Rb1, and pachymic acid (Figure 10A). Quantitative analysis showed that catalpol and rehmannioside were the major constituents derived from *Rehmannia glutinosa*, ginsenosides Rg1 and Rb1 from *Panax ginseng*, and pachymic acid from *Poria cocos* (Table 2). UPLC–QTOF–MS/MS further identified 30 compounds in QYG, including amino acids, organic acids, saccharides, saponins, flavonoids, and triterpenes (Supplementary Figure S2, Table S1). In serum, ginsenosides Rb1, Rh2, and B2 were detected at higher levels in the QYG group than in the Blank group (Figure 10B), indicating systemic exposure to ginseng-derived constituents after oral QYG administration.

**Figure 11:**
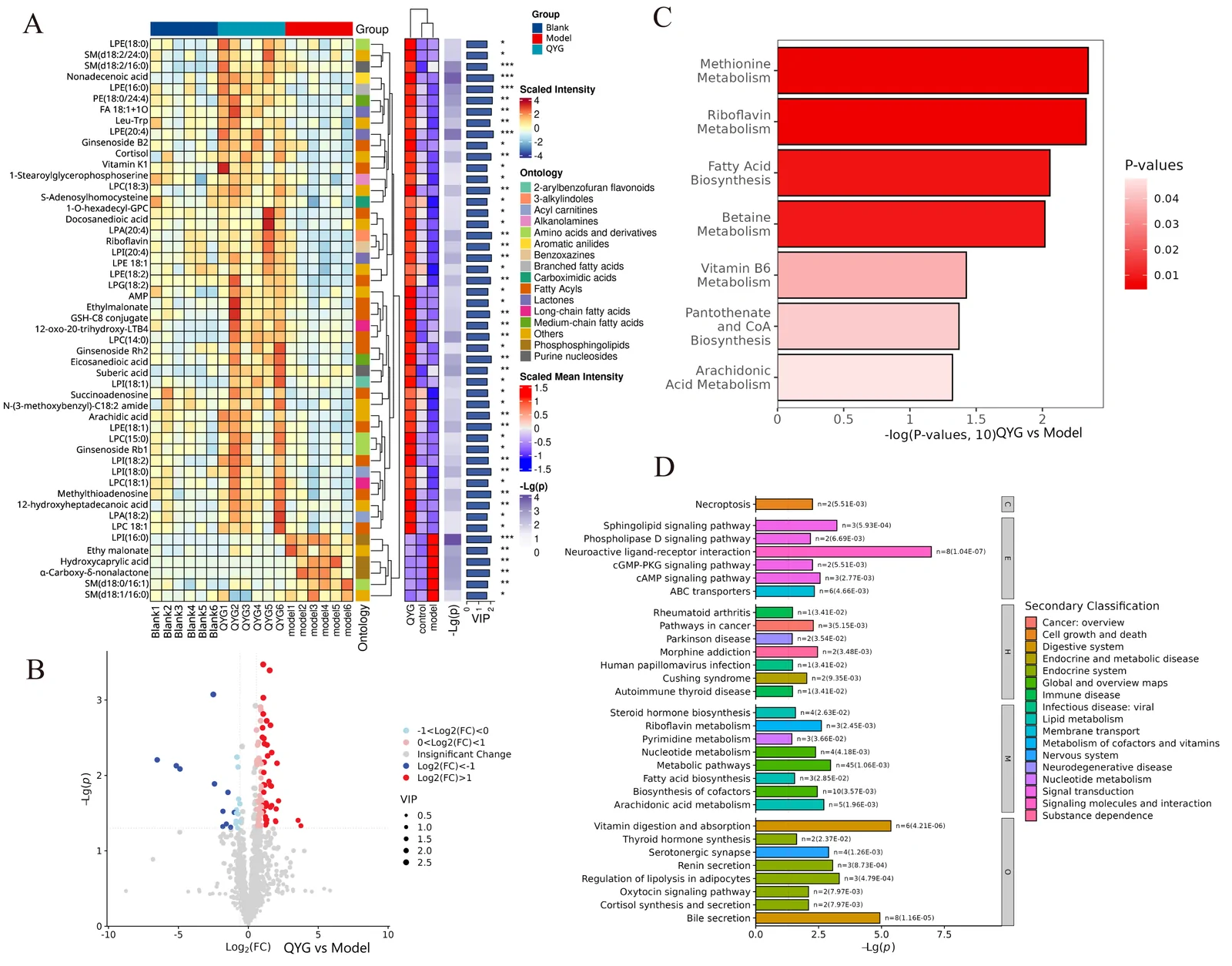
Hierarchical clustering and pathway enrichment analyses of differential serum metabolites in aged mice with UV-induced photoaging. (**A**) Hierarchical clustering heatmap of representative differential serum metabolites among the Blank, Model, and QYG groups. The right-side annotations indicate metabolite ontology, group-level scaled mean intensity, statistical significance, and VIP values. (**B**) Volcano plot showing the distribution of differential serum metabolites between the QYG and Model groups. Colors indicate fold-change categories and statistical significance as defined in the plot legend, and dot size represents VIP values. (**C**) Small Molecule Pathway Database (SMPDB) pathway enrichment analysis of differential metabolites between the QYG and Model groups. Bar length represents −log_10_(*p*), and color intensity indicates the corresponding *p*-value. (**D**) Kyoto Encyclopedia of Genes and Genomes (KEGG) pathway enrichment analysis of differential metabolites between the QYG and Model groups. Colors indicate secondary pathway classifications, and bar length represents −log_10_(*p*). Serum samples from six biological replicates per group were used for metabolomic analysis (*n* = 6).Statistical significance is indicated as * *p* < 0.05, ** *p* < 0.01, and *** *p* < 0.001.

Serum metabolomic profiling revealed distinct metabolic patterns among the Blank, Model, and QYG groups. The OPLS-DA score plot showed clear separation among the three groups, indicating that photoaging induction and QYG intervention markedly altered systemic metabolic status (Figure 10C). Volcano plot analysis identified significantly altered metabolites between the Model and QYG groups (Figure 11B), while hierarchical clustering of biologically plausible differential metabolites showed that the QYG group displayed a metabolic profile distinct from the Model group and partly closer to the Blank group (Figure 11A).

Differential metabolite analysis showed that QYG mainly regulated metabolites related to energy metabolism, fatty acid metabolism, acylcarnitine metabolism, and sphingolipid metabolism (Figure 10D and Figure 11A). Specifically, QYG increased citric acid and several dicarboxylic acids, including suberic acid, eicosanedioic acid, octadecanedioic acid, and tetradecanedioic acid. QYG also altered fatty acid oxidation-related metabolites, including 8-dodecenoylcarnitine, 9-decenoylcarnitine, undec-3-enoylcarnitine, 6-keto-decanoylcarnitine, 3,5-tetradecadiencarnitine, and O-adipoylcarnitine, together with fatty acid derivatives such as 10-hydroxydecanoate, hydroxycaprylic acid, 3-oxotetradecanoic acid, myristoleic acid, and palmitoleic acid. In addition, QYG decreased serum AMP and reduced sphingomyelin species, including SM(d18:2/16:0), SM(d18:1/16:0), and SM(d18:0/16:1). These results suggest that QYG remodeled serum metabolic signatures associated with the TCA cycle, fatty acid oxidation, mitochondrial substrate supply, energy status, and membrane lipid homeostasis.

Pathway enrichment analysis further showed that the differential metabolites were mainly involved in methionine metabolism, riboflavin metabolism, fatty acid biosynthesis, betaine metabolism, pantothenate and CoA biosynthesis, arachidonic acid metabolism, sphingolipid signaling, cGMP-PKG signaling, cAMP signaling, nucleotide metabolism, and bile secretion (Figure 11C,D). Together, these findings provide serum metabolic evidence supporting the involvement of energy and lipid metabolism remodeling in the anti-photoaging effects of QYG.

### 3.5. Multi-Omics Integration of the Gut–Skin Axis

**Figure 12:**
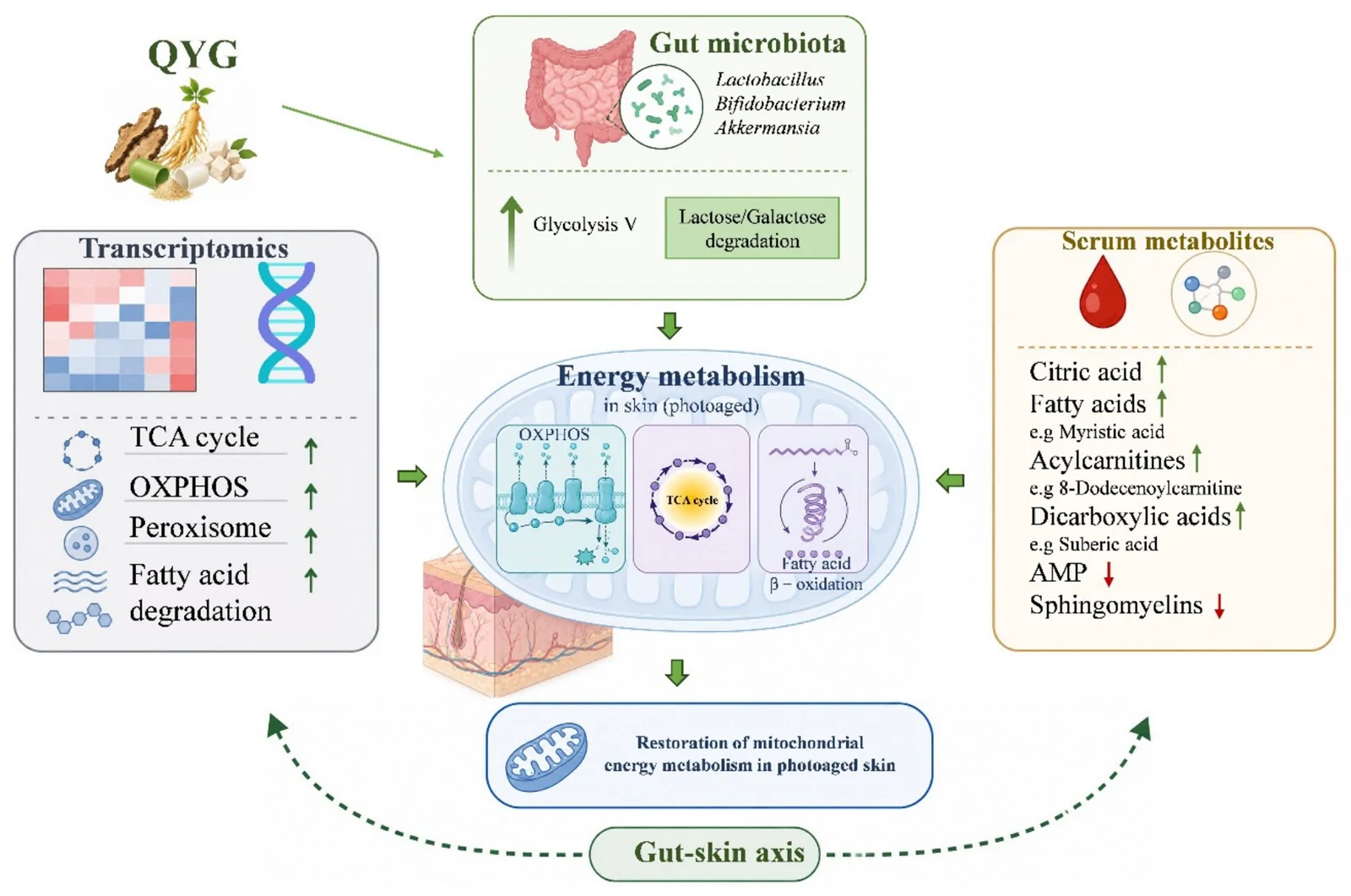
Multi-omics integration reveals QYG-associated restoration of mitochondrial energy metabolism through the gut–skin axis. Schematic summary integrating skin transcriptomics, gut microbiota analysis, and serum metabolomics in aged photoaged mice after QYG treatment. Skin transcriptomic analysis indicated enrichment of energy metabolism-related pathways, including the tricarboxylic acid (TCA) cycle, oxidative phosphorylation (OXPHOS), peroxisome, and fatty acid degradation. Gut microbiota analysis showed that QYG reshaped microbial composition, with increased *Lactobacillus*, *Bifidobacterium*, and *Akkermansia*, and altered predicted microbial metabolic functions, including glycolysis V and lactose/galactose degradation. Serum metabolomics further showed regulation of energy metabolism-related metabolites, including increased citric acid, fatty acids, acylcarnitines, and dicarboxylic acids, together with decreased AMP and sphingomyelins. These integrated findings suggest that QYG may alleviate skin photoaging by remodeling gut microbiota and systemic metabolism, thereby contributing to the restoration of mitochondrial energy metabolism in photoaged skin.

To systematically elucidate the multi-level mechanisms by which QYG alleviates UV-induced skin photoaging, we integrated the skin transcriptomic, gut microbiota, and serum metabolomic results. At the skin transcriptomic level, QYG treatment significantly enriched mitochondrial energy metabolism-related pathways in photoaged skin, including the tricarboxylic acid (TCA) cycle, oxidative phosphorylation, peroxisome, mitochondrial protein complex, and fatty acid degradation-related pathways (Figure 12). The TCA cycle and oxidative phosphorylation are central to mitochondrial ATP production and are closely associated with redox homeostasis and cellular metabolic adaptation [28,29]. In addition, fatty acid oxidation provides substrates for mitochondrial energy production, while dicarboxylic acids generated through fatty acid ω-oxidation can be further metabolized through β-oxidation (Ranea-Robles and Houten, 2023). These findings suggest that QYG may improve mitochondrial energy metabolism-related transcriptional programs in photoaged skin.

At the gut microbiota level, QYG selectively reshaped the intestinal microbial community. Although **α**-diversity was not restored to the Blank level, β-diversity, random forest analysis, and LEfSe analysis showed clear differences among the Blank, Model, and QYG groups. QYG enriched *Lactobacillus*-, *Bifidobacterium*-, and *Akkermansia*-associated taxa and altered predicted microbial carbohydrate-utilization functions, including glycolysis V and lactose/galactose degradation I (Figure 12). HUMAnN2 stratification further indicated that the increased glycolysis V pathway was mainly contributed to by *Bifidobacterium*-, *Lactobacillus*-, OD1-, and *Lachnospiraceae*-associated taxa, whereas lactose and galactose degradation I was predominantly contributed to by *Lactobacillus*-associated taxa. Previous studies have shown that gut microbiota and microbiota-derived metabolites can influence host metabolic health and systemic energy homeostasis [1,30,31]. Therefore, QYG-induced alterations in microbial carbohydrate utilization may provide a microbiota-level basis for downstream metabolic remodeling.

At the serum metabolomic level, QYG remodeled energy- and lipid-related metabolic signatures, including citric acid, dicarboxylic acids, acylcarnitines, fatty acid derivatives, AMP, and sphingomyelins (Figure 12). These metabolites were mainly associated with the TCA cycle, fatty acid oxidation, mitochondrial substrate supply, energy status, and lipid homeostasis. In particular, increased citric acid, dicarboxylic acids, acylcarnitines, and fatty acid derivatives, together with reduced AMP and sphingomyelin species, supported systemic metabolic remodeling related to mitochondrial energy metabolism and lipid metabolic balance.

Collectively, these results suggest that oral QYG may alleviate photoaging by linking gut microbiota remodeling, systemic metabolic regulation, and mitochondrial energy metabolism restoration in skin through the gut–skin axis (Figure 12).

### 3.6. Mitochondrial Function and AMPK-Associated Energy Metabolism Markers in MDFs

**Figure 13:**
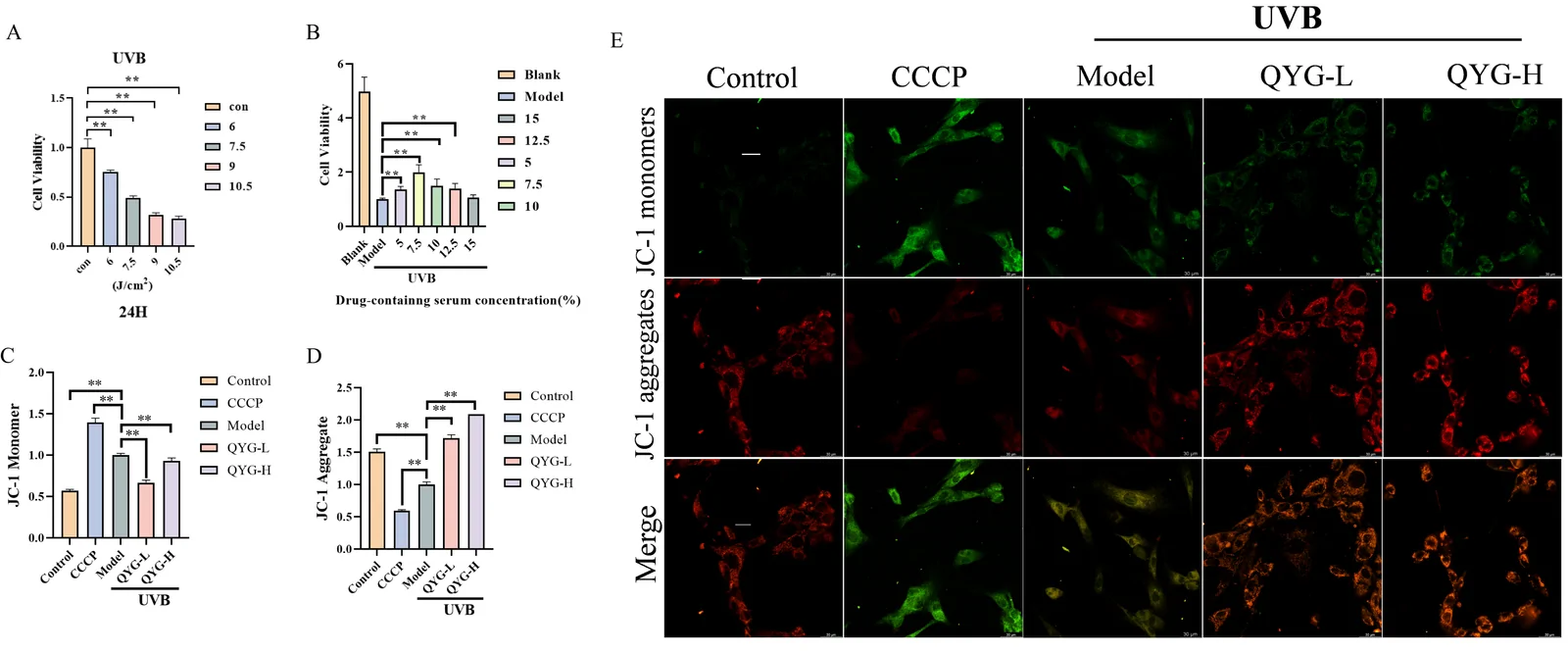
Establishment of the UVB-induced cellular photoaging model and effects of QYG-containing serum on mitochondrial membrane potential in primary mouse dermal fibroblasts. (**A**) UVB dose screening using the CCK-8 assay. A dose of 7.5 J/cm^2^, which reduced cell viability by approximately 50%, was selected for subsequent experiments (*n* = 6). (**B**) Screening of QYG-containing serum concentrations using the CCK-8 assay. Concentrations of 7.5% and 10% were selected as the low-dose (QYG-L) and high-dose (QYG-H) treatments, respectively (*n* = 6). (**C**,**D**) Quantification of JC-1 monomer and JC-1 aggregate fluorescence, respectively (*n* = 3). (**E**) Representative fluorescence images of JC-1 monomers, JC-1 aggregates, and merged signals. Green fluorescence indicates JC-1 monomers, whereas red fluorescence indicates JC-1 aggregates. Carbonyl cyanide m-chlorophenyl hydrazone (CCCP) was used as a positive control for mitochondrial membrane depolarization. Data are presented as the mean ± SEM. ** *p* < 0.01.

To validate the energy metabolism-related findings from the multi-omics analysis, UVB-injured primary mouse dermal fibroblasts were used to evaluate mitochondrial function, oxidative stress, cellular senescence, and AMPK-associated markers. First, the optimal UVB irradiation dose was determined using the CCK-8 assay. Cell viability decreased significantly in a dose-dependent manner, and 7.5 J/cm^2^ UVB irradiation reduced viability by approximately 50%, which was selected for subsequent experiments (Figure 13A, *n* = 6, *p* < 0.01). Screening of drug-containing serum showed that 7.5% and 10% QYG-containing serum exhibited no significant cytotoxicity, and these concentrations were designated as the QYG-L and QYG-H treatment groups, respectively (Figure 13B, *n* = 6, *p* < 0.01).

Mitochondrial membrane potential was evaluated using JC-1 staining. Compared with the model group, the QYG treatment groups showed significantly reduced JC-1 monomer fluorescence and increased JC-1 aggregate fluorescence, indicating attenuation of UVB-induced mitochondrial depolarization (Figure 13C–E, *n* = 3, *p* < 0.01).

**Figure 14:**
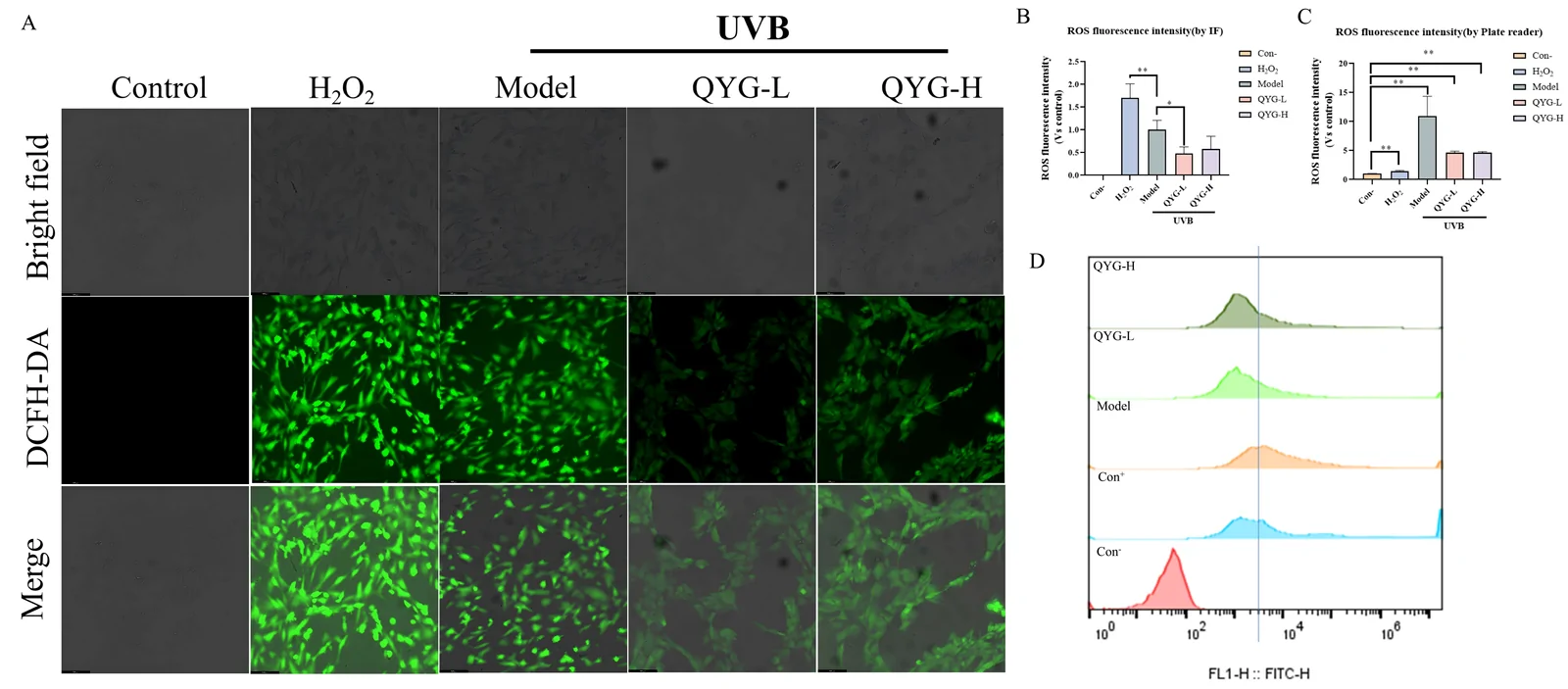
QYG-containing serum reduces UVB-induced intracellular ROS accumulation in primary mouse dermal fibroblasts. (**A**) Representative bright-field, DCFH-DA fluorescence, and merged images of intracellular ROS. H_2_O_2_ was used as a positive control for ROS generation. Scale bar = 100 μm. (**B**) Quantification of DCFH-DA fluorescence intensity from fluorescence images using ImageJ software (*n* = 3). (**C**) Quantification of intracellular ROS fluorescence using a microplate reader at excitation/emission wavelengths of 488/525 nm (*n* = 6). (**D**) Representative flow cytometric histograms of DCFH-DA fluorescence in the Control, H_2_O_2_, Model, QYG-L, and QYG-H groups. Data are presented as the mean ± SEM. * *p* < 0.05 and ** *p* < 0.01.

Intracellular ROS levels were further assessed using the DCFH-DA fluorescent probe. Representative fluorescence images showed increased DCFH-DA fluorescence in the Model group, whereas QYG treatment reduced intracellular ROS fluorescence (Figure 14A). Quantitative analysis with ImageJ (Figure 14B, *n* = 3) and a microplate reader (Figure 14C, *n* = 6) confirmed these reductions (*p* < 0.01). Flow cytometry further validated the decrease in ROS fluorescence intensity after QYG treatment (Figure 14D).

**Figure 15:**
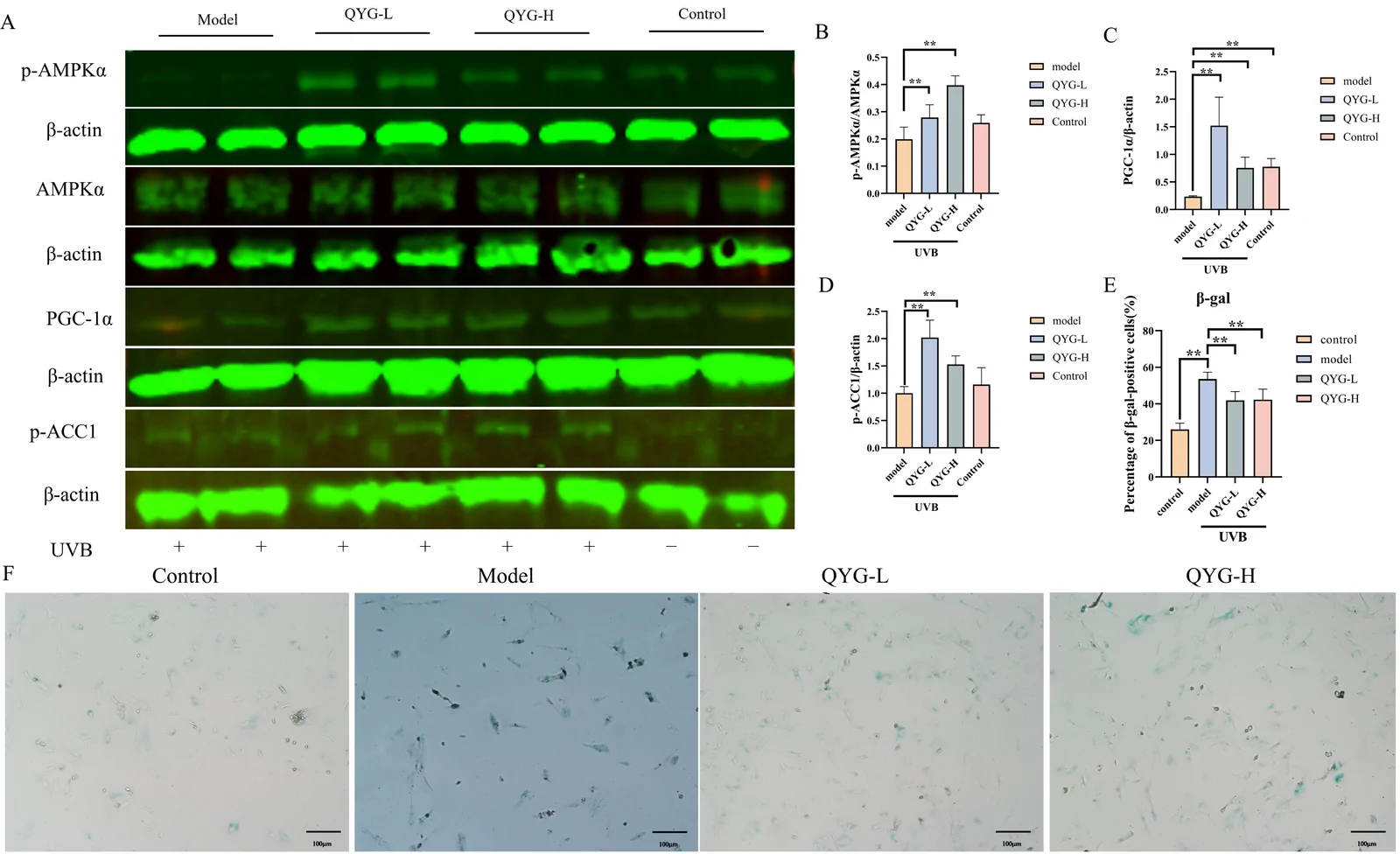
QYG-containing serum modulates AMPK-associated proteins and alleviates UVB-induced cellular senescence in primary mouse dermal fibroblasts. (**A**) Representative Western blot images of phosphorylated AMP-activated protein kinase α (p-AMPKα), total AMPKα, peroxisome proliferator-activated receptor gamma coactivator 1α (PGC-1α), phosphorylated acetyl-CoA carboxylase 1 (p-ACC1), and β-actin. (**B**–**D**) Quantification of the p-AMPKα/AMPKα ratio, PGC-1α/β-actin ratio, and p-ACC1/β-actin ratio, respectively (*n* = 3). (**E**) Percentage of senescence-associated β-galactosidase (SA-β-gal)-positive cells (*n* = 5). (**F**) Representative images of SA-β-gal staining. Scale bar = 100 μm. Data are presented as the mean ± SEM. ** *p* < 0.01.

Western blot analysis showed that UVB irradiation reduced the p-AMPKα/AMPKα ratio and PGC-1α protein level compared with the Control group. QYG treatment significantly increased the p-AMPKα/AMPKα ratio, PGC-1α protein level, and p-ACC1 protein level compared with the Model group (Figure 15A–D, *n* = 3, *p* < 0.05 or *p* < 0.01).

SA-β-gal staining revealed that the proportion of senescent cells was significantly elevated in the model group, whereas both QYG-L and QYG-H treatments reduced the percentage of SA-β-gal-positive cells (Figure 15E,F, *n* = 5, *p* < 0.01).

## 4. Discussion

### 4.1. Food–Medicine Homologous QYG

QYG is a classical food–medicine homologous oral formula composed of *Rehmannia glutinosa*, *Panax ginseng*, and *Poria cocos*. Chemical profiling confirmed representative constituents of the three botanical materials, providing a quality basis for evaluating QYG as a dietary intervention for skin photoaging.

In young and aged photoaged mice, QYG improved visible and histological features of skin injury, including epidermal thickening, collagen disorganization, oxidative stress, inflammation, extracellular matrix degradation, and hyaluronic acid loss. These results indicate that QYG exerted multi-level protection against photoaging-related skin damage.

The skin-protective activity of QYG may be related to the integrated actions of its food–medicine homologous botanical ingredients after oral administration. Ginsenosides have been reported to regulate mitochondrial function, oxidative stress, inflammation, and energy metabolism. For example, ginsenoside Rb1 has been shown to protect against acute myocardial ischemic injury by regulating metabolomic profiles and AMPKα-dependent mitophagy, and to improve energy metabolism and mitochondrial function after spinal cord injury [32,33]. Catalpol from *Rehmannia glutinosa* has been reported to protect against D-galactose-induced mitochondrial dysfunction and energy metabolism impairment [34,35]. Poria-derived polysaccharides and triterpenoids have also been associated with anti-inflammatory effects, extracellular matrix regulation, and gut microbiota modulation [36,37]. Therefore, the anti-photoaging effects of QYG are more likely to reflect the integrated actions of multiple orally administered food-derived botanical constituents rather than the activity of a single compound.

The detection of serum-exposed ginsenosides further supports the systemic availability of selected QYG constituents after oral administration, although the overall activity of QYG should still be interpreted at the formula level.

### 4.2. Gut Microbiota Remodeling by Dietary QYG

The gut–skin axis has received increasing attention as a systemic regulatory route linking intestinal microbiota, immune-metabolic homeostasis, and skin physiology. UV exposure and aging can alter the gut microbial ecosystem, while gut microbiota and microbiota-derived metabolites are increasingly recognized as important regulators of host metabolic health [1,22]. In the present study, QYG did not simply restore **α**-diversity to the Blank level. Instead, it selectively reshaped gut microbial community structure, as indicated by β-diversity separation, random forest analysis, and LEfSe results. This distinction is important because **α**-diversity alone should not be regarded as a direct indicator of microbiome health; microbiota-directed interventions may exert biological effects by selectively enriching or suppressing specific taxa rather than by broadly increasing overall diversity.

Consistent with this concept, QYG enriched several health-associated or probiotic-related taxa, including *Lactobacillus*, *Bifidobacterium*, and *Akkermansia muciniphila*. The term “probiotic” should be used cautiously because it is defined at the strain level and requires evidence that live microorganisms confer a health benefit when administered in adequate amounts [38]. Therefore, these taxa are more appropriately described as health-associated or probiotic-related taxa rather than directly as probiotics. *Lactobacillus* and *Bifidobacterium* are classical probiotic-associated genera with long-standing relevance to intestinal health, whereas *Akkermansia muciniphila* is widely regarded as a next-generation beneficial microorganism associated with gut barrier function and metabolic regulation [25,26]. The enrichment of these taxa suggests that QYG may create a microbial environment favorable for host metabolic regulation.

Predicted functional analysis further suggested that QYG altered microbial metabolic potential. Compared with the Model group, QYG increased the predicted abundance of glycolysis V and maintained high *Lactobacillus*-associated lactose and galactose degradation I. HUMAnN2 stratification showed that the increased glycolysis V pathway was mainly contributed to by *Bifidobacterium*-, *Lactobacillus*-, OD1-, and *Lachnospiraceae*-associated taxa. These results suggest that QYG-induced microbiota remodeling was accompanied by changes in microbial carbohydrate utilization and glycolysis-related functions. Considering that QYG contains abundant plant-derived polysaccharides and other water-soluble constituents, this pattern is biologically plausible, because dietary carbohydrates and plant-derived polysaccharides can directly interact with gut microbes and influence microbial metabolic outputs [3].

Although short-chain fatty acids were not directly quantified, previous studies support that gut microbiota-derived metabolites can influence host energy metabolism and systemic homeostasis. Therefore, the observed microbial functional changes may provide an upstream basis for serum metabolic remodeling.

### 4.3. Mitochondrial Energy Metabolism and Skin Repair

A central finding of this study is that QYG-induced protection against photoaging converged on energy metabolism across the transcriptomic, microbial, metabolomic, and cellular levels. In skin transcriptomics, QYG significantly enriched pathways related to the TCA cycle, oxidative phosphorylation, peroxisome, mitochondrial protein complex, DNA replication, and fatty acid degradation. Mitochondria are central hubs of cellular metabolism, supporting ATP generation, redox homeostasis, biosynthetic precursor supply, and stress adaptation [27,29]. The TCA cycle and oxidative phosphorylation are closely linked to mitochondrial bioenergetics, and TCA cycle intermediates can also regulate broader cellular functions and disease-related processes [12,28].

The serum metabolomic results further supported the role of systemic metabolic regulation in the anti-photoaging effects of QYG. QYG increased citric acid, a key intermediate of the TCA cycle. TCA cycle metabolites are not only involved in mitochondrial energy production but also participate in broader cellular signaling, redox regulation, and stress adaptation [12]. QYG also increased several dicarboxylic acids, including suberic acid, eicosanedioic acid, octadecanedioic acid, and tetradecanedioic acid. Long-chain dicarboxylic acids are generated through fatty acid ω-oxidation and can be further metabolized through β-oxidation, reflecting changes in fatty acid oxidative metabolism [39]. In addition, QYG regulated multiple acylcarnitines, including 8-dodecenoylcarnitine, 9-decenoylcarnitine, undec-3-enoylcarnitine, 6-keto-decanoylcarnitine, 3,5-tetradecadiencarnitine, and O-adipoylcarnitine. Acylcarnitines are important intermediates related to mitochondrial fatty acid transport and β-oxidation and are widely used as metabolic indicators of fatty acid oxidation and mitochondrial energy metabolism [40].

Serum AMP was decreased after QYG treatment. Because AMPK senses cellular energy status through changes in adenine nucleotide balance, particularly AMP/ATP and ADP/ATP ratios, reduced serum AMP may reflect attenuation of an energy-stress-associated metabolic state in the Model group [41,42]. However, serum AMP does not directly represent intracellular AMP/ATP status; therefore, this result should be interpreted as supportive evidence of systemic energy metabolism remodeling rather than direct proof of AMPK activation. QYG also reduced sphingomyelin species such as SM(d18:2/16:0), SM(d18:1/16:0), and SM(d18:0/16:1). Sphingomyelins are major structural sphingolipids in mammalian membranes and participate in membrane organization and lipid signaling [43]. Abnormal sphingolipid accumulation has been linked to mitochondrial dysfunction and metabolic stress [29,44]. Therefore, the reduction in model-elevated sphingomyelin species suggests that QYG may contribute to the restoration of membrane lipid homeostasis, which may be relevant to mitochondrial function and skin barrier stability.

Together with the gut microbiota results, the serum metabolomic findings suggest that QYG-induced microbial remodeling was accompanied by systemic metabolic reprogramming. Because microbial metabolites such as SCFAs were not directly quantified, the relationship between gut microbiota and serum metabolic changes should be interpreted as an association rather than direct causality. Nevertheless, the consistency among microbial carbohydrate-utilization functions, serum energy-related metabolites, and skin transcriptomic energy pathways supports the gut–skin axis as a reasonable framework for explaining the systemic anti-photoaging effects of QYG.

The cellular validation further supported the energy metabolism-related interpretation of the multi-omics findings. In UVB-injured primary mouse dermal fibroblasts, QYG-containing serum restored mitochondrial membrane potential, reduced intracellular ROS accumulation, decreased SA-β-gal-positive cells, and regulated AMPK-associated energy metabolism markers, including increased p-AMPKα and PGC-1α and decreased p-ACC1. AMPK is a conserved energy sensor that coordinates metabolic adaptation under cellular stress, while PGC-1α is a major regulator of mitochondrial biogenesis, oxidative metabolism, and antioxidant defense [41,42,45,46]. The increased p-AMPKα and PGC-1α levels therefore suggest that QYG-containing serum may enhance cellular energy-sensing and mitochondrial regulatory responses. In parallel, the decrease in p-ACC1 indicates that QYG also affected lipid metabolism-related signaling. Because p-ACC1 changed in the opposite direction to p-AMPKα and PGC-1α, this result should be interpreted as evidence of altered lipid metabolic regulation rather than direct evidence of canonical AMPK–ACC activation.

Together, these findings support a multi-level mechanism involving gut microbiota remodeling, systemic metabolic regulation, and mitochondrial energy metabolism restoration in photoaged skin.

## 5. Conclusions

This study demonstrated that QYG, a food–medicine homologous oral formula, protected against combined D-galactose- and UVA/UVB-induced skin photoaging in both young and aged mice. Oral QYG improved visible skin damage, epidermal thickening, collagen disorganization, oxidative stress, inflammatory responses, extracellular matrix degradation, and hyaluronic acid loss. Chemical profiling confirmed representative constituents from *Rehmannia glutinosa*, *Panax ginseng*, and *Poria cocos*, while serum-detected ginsenosides supported systemic exposure after oral administration.

Integrated multi-omics analysis revealed that the protective effects of QYG were associated with coordinated regulation of gut microbiota, serum metabolism, and mitochondrial energy metabolism. Skin transcriptomics showed enrichment of mitochondrial energy metabolism-related pathways; gut microbiota analysis indicated selective remodeling of *Lactobacillus*-, *Bifidobacterium*-, and *Akkermansia*-associated taxa; and serum metabolomics revealed alterations in energy- and lipid-metabolism-related metabolites. Cellular experiments further confirmed that QYG-containing serum improved mitochondrial function, reduced oxidative stress and cellular senescence, and regulated AMPK/PGC-1α-associated energy metabolism markers. Collectively, these findings provide experimental evidence supporting QYG as a promising food–medicine homologous oral botanical intervention for the dietary management of skin photoaging.

## Figures and Tables

**Table 1:** Kong and Bisset Skin Wrinkle Rating Scale.

Grade	Characteristics
0	No visible wrinkles or looseness; normal skin texture
1	Slight fine desquamation
2	A few shallow wrinkles
3	Many shallow wrinkles
4	Rough skin, a few deep wrinkles
5	Many deep wrinkles
6	Severe wrinkles with serious skin damage

**Table 2:** Quantitative results of QYG by HPLC.

Ingredients	Linear	R^2^	Range (mg/mL)	tR	AR	Content (%)
Catalpol	y = 9.5360x − 0.0752	0.9999	0.165–2.105	5.900	6.660	1.41 ± 0.003
Rehmannioside	y = 1.4966x − 0.0019	0.9999	0.135–2.139	13.770	1.526	2.04 ± 0.007
Ginsenoside Rg1	y = 4.2585x − 0.0157	0.9999	0.121–2.117	20.007	0.415	0.20 ± 0.001
Ginsenoside Rb1	y = 3.0483x − 0.0185	0.9997	0.104–2.138	21.970	0.327	0.23 ± 0.001
Pachymic Acid	y = 5.0128x + 0.0582	0.9926	0.148–1.784	42.070	0.153	0.03 ± 0.001

## Data Availability

The original contributions presented in this study are included in the article/supplementary material. Further inquiries can be directed to the corresponding authors.

## Supplementary Materials

The following supporting information can be downloaded at: https://www.mdpi.com/article/10.3390/foods15162824/s1, Figure S1: Effect of QYG on oxidative stress, extracellular matrix components, and inflammatory gene expression in the UV- and D-galactose-induced skin aging model of young mice; Figure S2: Raw LC–MS full scan of QYG extract in (A) positive and (B) negative ion modes; Table S1: Qualitative identification of 30 compounds in QYG by HPLC-Q-TOF-MS/MS; Table S2: Primer Sequences.

## Abbreviations

The following abbreviations are used in this manuscript:

ACC	Acetyl-CoA Carboxylase
AMP	Adenosine Monophosphate
AMPK	AMP-activated Protein Kinase
CAT	Catalase
DCFH-DA	2’,7’-Dichlorodihydrofluorescein Diacetate
DEG	Differentially Expressed Gene
ELISA	Enzyme-Linked Immunosorbent Assay
FGF-23	Fibroblast Growth Factor 23
GSH-Px	Glutathione Peroxidase
HA	Hyaluronic Acid
H&E	Hematoxylin and Eosin
HPLC	High-Performance Liquid Chromatography
IL	Interleukin
KEGG	Kyoto Encyclopedia of Genes and Genomes
LEfSe	Linear Discriminant Analysis Effect Size
MDA	Malondialdehyde
MDFs	Mouse Dermal Fibroblasts
MS	Mass Spectrometry
MMP	Matrix Metalloproteinase
n	Number of animals or samples
OTU	Operational Taxonomic Unit
OXPHOS	Oxidative Phosphorylation
PCA	Principal Component Analysis
PGC-1α	Peroxisome Proliferator–Activated Receptor Gamma Coactivator 1-α
QYG	Qiongyu Gao
ROS	Reactive Oxygen Species
SA-β-gal	Senescence-Associated β-Galactosidase
SCFA	Short-Chain Fatty Acid
SOD	Superoxide Dismutase
TCA	Tricarboxylic Acid
TNF	Tumor Necrosis Factor
UV/UVR	Ultraviolet/Ultraviolet Radiation
