# Supplementary material for: Food–Medicine Homologous Qiongyu Gao Attenuates Skin Photoaging by Remodeling Gut Microbiota and Restoring Mitochondrial Energy Metabolism
Source: Foods. 2026 Aug 13;15(16):2824. doi: 10.3390/foods15162824 (PMC13512444; doi:10.3390/foods15162824)
Supplement: Supplementary file 1 [file foods-15-02824-s001.zip › foods-4436046-supplementary.pdf]

Fig. S.1

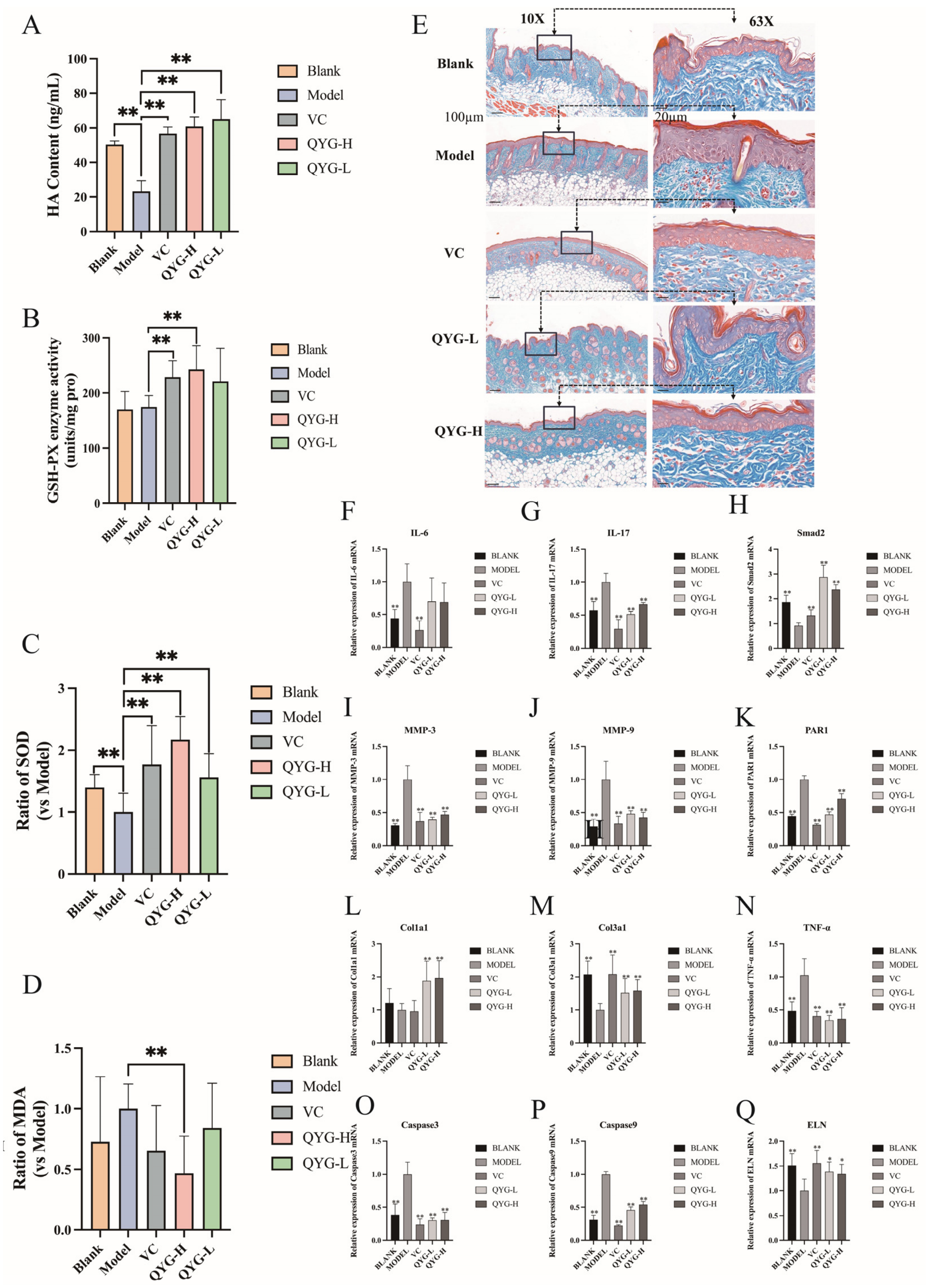

Figure S1. Effect of QYG on oxidative stress, extracellular matrix components, and inflammatory

gene expression in UV and D-galactose-induced skin aging model of young mice. (A–D) Quantitative biochemical measurements of skin antioxidant and matrix-related indicators. (A) Skin hyaluronic acid (HA) content.(B) Glutathione peroxidase (GSH-Px) enzyme activity. (C) Superoxide dismutase (SOD) activity (normalized to model group).(D) Malondialdehyde (MDA) level (normalized to model group).All data are shown as mean ± SD (n = 6). Statistical analysis was performed using one-way ANOVA; $^*p < 0.05$, ** $p < 0.01$.(E) Representative images of Masson's trichrome staining of dorsal skin tissue sections (10× and 63× magnification). Blue-stained collagen fibers indicate extracellular matrix integrity. QYG treatment restored collagen density compared to the model group.(F–Q) RT-qPCR analysis of genes involved in inflammation, matrix remodeling, and apoptosis in mouse dorsal skin.(F) IL-6, (G) IL-17, (H) Smad2, (I) MMP-3, (J) MMP-9, (K) PAR1, (L) Col1a1, (M) Col3a1, (N) TNF-α, (O) Caspase-3, (P) Caspase-9, (Q) ELN (elastin).Gene expression was normalized to β-actin and presented as fold change relative to blank control. All data are mean ± SD (n = 6); statistical significance: * $p < 0.05$, ** $p < 0.01$ versus model group.

Fig. S.2

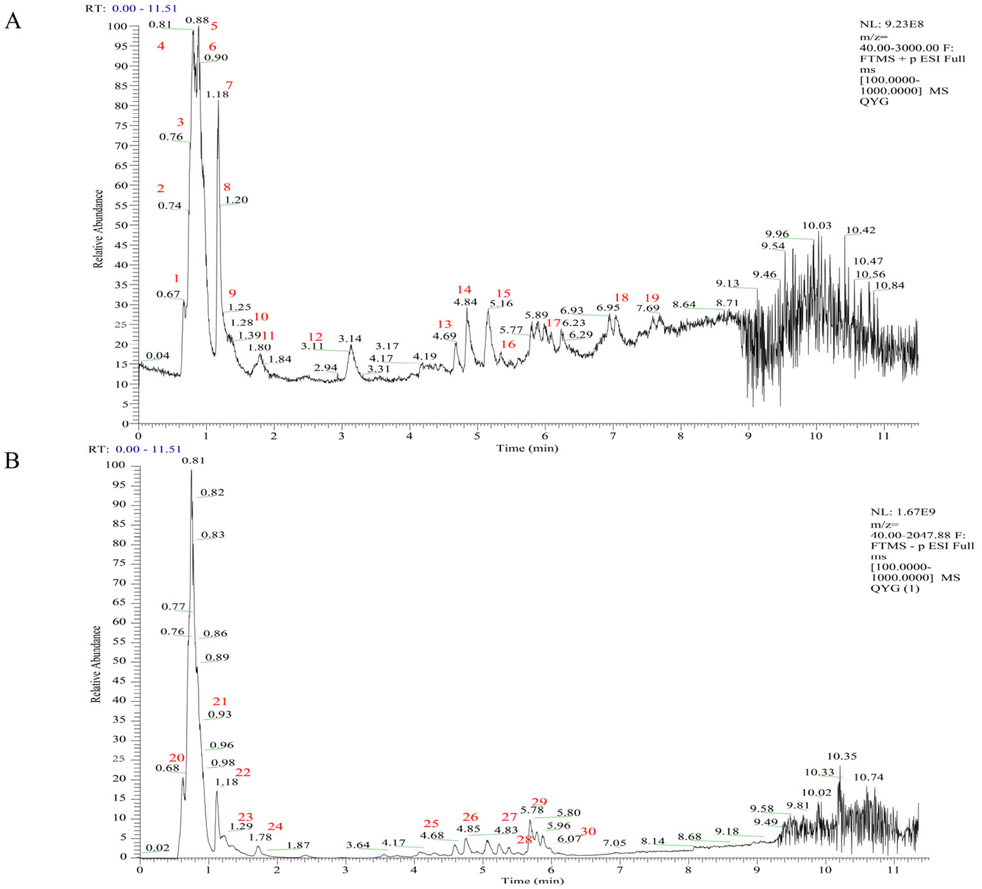

Figure S2. Raw LC–MS full scan of QYG extract in (A) positive and (B) negative ion modes.

Table S1. Qualitative identification of 30 compounds in QYG by HPLC-Q-TOF-MS/MS.

Number	RT(s)	Ion mode	Identification	m/z	Formula	ppm	class	Area (Max.)
1	43.8	[M+H] +	L-Arginine	175.1186	C6H14N4O2	1.98	Carboxylic acids and derivatives	1001204166
2	45.5	[M+NH4] +	Dephospho-CoA	705.1728	C21H35N7O13P2S	14.03545358	Purine nucleotides	304822852.1
3	49.4	[M+NH4] +	Raffinose	522.1975	C18H32O16	10.25	Organooxygen compounds	447484568
4	49.7	[M+H] +	1,3,5-Trihydroxybenzene	127.0384	C6H6O3	4.479	Benzenoids	100401390.2
5	49.7	[M+H] +	L-Proline	116.07	C5H9NO2	5.185	Organic acids and derivatives	215944287.9
6	70.5	[M-NH3+H] +	N-Succinyl-L, L-2,6-diaminopimelate	274.0927	C11H18N2O7	2.104	Carboxylic acids and derivatives	562953536.2
7	70.5	[M+H] +	L-Tyrosine	182.0791	C9H11NO3	11.347	Carboxylic acids and derivatives	277323575.5
8	73.8	[M+H] +	Adenosine	268.1063	C10H13N5O4	8.481	Purine nucleosides	592683682.5
9	73.9	[M+H] +	N-(2-hydroxymethyl-3-chloro-4-hydroxyphenyl)anthranilic acid	294.1568	C14H12ClNO4	7.067	Benzene and substituted derivatives	852664383
10	77.5	[M+H] +	L-Leucine	132.1031	C6H13NO2	9.082	Carboxylic acids and derivatives	656414946.2
11	102.6	[M+H] +	L-Phenylalanine	166.0868	C9H11NO2	3.305	Carboxylic acids and	270293147.2

							derivatives	
12	182	[M+H] +	L-Tryptophan	205.0969	C11H12N2O2	1.217	Organoheterocyclic compounds	35147468.5
13	280.8	[M+H] +	Coumaroylquinic acid	339.1071	C16H18O8	1		35812067.84
14	294.3	[M-NH3+H] +	Pyridoxine	153.055	C8H11NO3	2.502	Organoheterocyclic compounds	2142067.7
15	310.3	[M-H2O+H] +	N-Acetyl-D-glucosamine	204.087	C8H15NO6	1.742296	Organic oxygen compounds	8763645.2
16	315.3	[M+NH4] +	Arbutin	290.1225	C12H16O7	3.184	Organic oxygen compounds	7733605.0
17	365.6	[M-H2O+H] +	β-Amyrenonol	423.3601	C30H48O2	4.777		412453691.8
18	422.6	[M+NH4] +	Heptadecanoic acid	288.2908	C17H34O2	3.844	Fatty Acyls	1889112737
19	461.8	[M+Na] +	2-IMINIOPROPANOATE	110.0214	C3H5NO2	1.384275241	Organonitrogen compounds	1994484063
20	40.9	[M-H]-	DL-Malic acid	133.0146	C4H6O5	2.66	Hydroxy acids and derivatives	2862089945
21	57	[M+HCOO]-	D-Gal alpha 1->6D-Gal alpha 1->6D-Glucose	549.1669	C18H32O16	0.602	Organooxygen compounds	665087395.7
22	71	[M+HCOO]-	Catalpol	407.1244	C15H22O10	12.049		579228223.8
23	77.5	[M-H2O-H]-	Sebacic acid	183.104	C10H18O4	7.311	Fatty Acyls	858454203.8

24	108.1	[M-H]-	Isocitric acid	191.0566	C6H8O7	2.571	Organic acids and derivatives	4994129.2
25	281.4	[M+HCOO]-	2-Hydroxypseudobaptigenin	343.0482	C16H10O6	6.58922		5049335.0
26	293.9	[M-H]-	Caproic acid	115.0037	C6H12O2	0.157	Lipids and lipid-like molecules	19070045.1
27	309.9	[M+HCOO]-	Ginsenoside A2	845.4953	C42H72O14	5.813		220405073.5
28	320.3	[M-H]-	Ginsenoside Ro	955.5017	C48H76O19	11.431	Lipids and lipid-like molecules	54399088.2
29	324.2	[M+HCOO]-	borneol	199.134	C10H18O	0.19394		2281447.8
30	365.7	[M+HCOO]-	Ginsenoside RG2	829.5028	C42H72O13	8.837		77264714.05

Table S2. Primer Sequence

Gene		Primer sequences (5'-3')
β-ACTIN	Forward	TGCTGTCCCTGTATGCCTCTG
	Reverse	TGATGTCACGCACGATTTCC
MMP-3	Forward	GGAAATCAGTTCTGGGCTATACGAGG
	Reverse	CCAACTGCGAAGATCCACTGAAGAAG
TNF-α	Forward	ACGGCATGGATCTCAAAGAC
	Reverse	GTGGGTGAGGAGCACGTAGT
MMP-9	Forward	CGTGGGTCGATTCCAAACCT
	Reverse	GTACACGCGGGTGAAGGTGA
IL-6	Forward	TTCCATCCAGTTGCCTTCTT
	Reverse	ATTTCCACGATTTCCCAGAG
Smad2	Forward	TCCGGCTGAACTGTCTCCTA
	Reverse	CTGTGACGCATGGAAGGTCT
Col1a1	Forward	GCGCTAAAGGTGCCAATGGT
	Reverse	ACCAGGTTCACCACTGTTAC
Col3a1	Forward	TCAACCAGTGCAAGTGACCA
	Reverse	GCTGGTGAATGAGTATGACCGT
IL-17A	Forward	CAGACTACCTCAACCGTTCCAC
	Reverse	TCCAGCTTTCCCTCCGCATTGA
PAR1	Forward	GCAGGCCAGAATCAAAAGCAACAAATGC
	Reverse	TCCTCATCCTCCCAAAATGGTTCA
Caspase3	Forward	GGAGTCTGACTGGAAAGCCGAA
	Reverse	CTTCTGGCAAGCCATCTCCTCA
Caspase9	Forward	TGCCC TTGCCTCTGAGTAGT
	Reverse	AACAAAGAAACGCCCACAAC
Elastin(Eln)	Forward	AAATATGGTGCTGCTGGCCTT
	Reverse	AAAGTCCAAAGCCAGGTCTTG
